# Boron from net charge acceptor to donor and its effect on hydrogen uptake by novel Mg-B-electrochemically synthesized reduced graphene oxide

**DOI:** 10.1038/s41598-021-90531-w

**Published:** 2021-05-26

**Authors:** Marla V. V. Satya Aditya, Srikanta Panda, Sankara Sarma V. Tatiparti

**Affiliations:** Department of Energy Science & Engineering, Indian Institute of Technology Bombay, Mumbai, 400076 India

**Keywords:** Hydrogen storage, Hydrogen storage materials

## Abstract

Hydrogen uptake (H-uptake) is studied in ball milled Mg-B-electrochemically synthesized reduced graphene oxide (erGO) nanocomposites at *P*_H2_ ≈ 15 bar, ~ 320 °C. *B/C* (weight ratio): 0, ~ 0.09, ~ 0.36, ~ 0.90 are synthesized maintaining erGO≈10wt %. B occupies octahedral interstices within Mg unit cell—revealed by electron density maps. Persistent charge donations from Mg and B to C appear as Mg-C (~ 283.2 eV), B-C (~ 283.3–283.9 eV) interactions in C-1s core X-ray photoelectron spectroscopy (XPS) at all *B/C*. At *B/C* > 0.09, charge reception by B from Mg yields Mg-B interaction. This net charge acceptor role of B renders it electron-rich and does not alter Mg unit cell size significantly. Despite charge donation to both C and B, the Mg charge is <  + 2, resulting in long incubation times (> 5 h) at *B/C* > 0.09. At *B/C*≈0.09 the minimal Mg-B interaction renders B a charge donor, resulting in Mg-B repulsion and Mg unit cell expansion. Mg-C peak shift to lower binding energies (C-1s XPS), decreases incubation time to ~ 2.25 h and enhances H-uptake kinetics. Various atomic interactions influence the reduction of incubation time in H-uptake and increase its kinetics in the order: (Mg → C; B → C)_*B/C*≈0.09, *B*: donor_ > (Mg → C)_*B/C*=0_ > (ternary Mg → B → C)_*B/C*>0.09, *B*: acceptor_.

## Introduction

Magnesium (hydride) is a promising material for hydrogen storage due to its high gravimetric capacity (~ 7.6 wt %) and its abundance (as oxide)^1^. However, its poor kinetics of hydrogen uptake (H-uptake) and release pose significant challenges for its use as on-board hydrogen storage material for automobile applications^2,3^. H-uptake by Mg suffers from the presence of incubation time during which Mg cannot absorb any hydrogen^4^. For example, Mg failed to hydrogenate at *P*_H2_ = 10 bar and 300 °C^4^. At 400 °C, H-uptake started only after 30 min of the commencement of the experiment^4^.

Nanosizing and catalyst addition can enhance the H-uptake/release kinetics significantly^5,6^. In a study, decreasing the particle size of MgH_2_ to ~ 12 nm by ball milling resulted in a four-fold decrease in the H-uptake/release time at *P*_H2_ ≈ 0.1 bar and ~ 350 °C^7^. For on-board hydrogen storage applications, the weight of the material needs to be kept minimal. The light weight, 2-dimensional carbon based materials such as reduced graphene oxide (rGO) are widely studied as catalysts, supports and confining agents^8^. They can enhance kinetics, and inhibit the agglomeration and surface oxidation of the nanosized Mg(H_2_) particles^9^. In a recent study, Liu et al.^10^ reported that 5 wt % of rGO can result in the desorption of ~ 6.3 wt% hydrogen from MgH_2_ in 80 min. Boron (B), which is even lighter than C, can also catalyze H-release in Mg-based materials. However, mostly, its catalytic effects have been realized only indirectly. For example, Mg(BH_4_)_2_ renders H-release in stages with the formation of an intermediate compound (MgB_12_H_12_) and accelerates the desorption process upon in-situ MgB_2_ formation^11^. This in-situ formation of MgB_2_ occurs even during desorption from hydride mixtures such as LiBH_4_-MgH_2_ and catalyzes H-release by MgH_2_^12^. Theoretical studies on B inclusion in MgH_2_ reported lattice changes and increase in H–H distance^13^. Binary Mg-B, B-rGO and Mg-rGO systems were studied in the past^9,10,14–16^. B-doped graphene improves enthalpy of H-release in MgH_2_^17^. Nevertheless, the investigations on Mg(H_2_)-B-rGO ternary system are in their early stages, particularly during H-uptake. Moreover, the charge transfer role of B and its impact on the hydrogen storage by Mg-rGO system are not well known from literature.

Most of the studies on hydrogen storage by MgH_2_-based materials are focussed on H-release rather than H-uptake. Some scientific reasons for this are (i) uncertainty involved in experimentally identifying the lattice positions of H, any elemental additions etc. in the crystalline hydrogen-hosts upon H-uptake; this further renders (ii) the investigation of various atomic interactions among the components difficult; (iii) such uncertainties in structural investigations can lead to erroneous estimation of enthalpy and entropy changes. This is particularly true in the case of composite systems such as Mg(H_2_)-B-rGO, where both Mg and C (from rGO) can, synergistically, form bonds with H^9,18^. Our earlier study on H-uptake and release by Mg-rGO demonstrates that along with Mg, C also forms bond with H due to charge (electron) transfer from Mg to C^18^. Practically, the H-uptake studies are less appealing than their H-release counterparts for reporting, since H-uptake involves long incubation times^4^. Nevertheless, hydrogen storage involves both H-uptake and release and the studies on the former are indispensable.

In the present study H-uptake by the novel Mg-B-erGO nanocomposites at *P*_H2_ ≈ 15 bar and ~ 320 °C is reported. The erGO is a novel electrochemically synthesized rGO, which we reported in our recent study, that contains fewer functional groups than rGO prepared by the modified Hummer’s method^19^. Here, Mg, B and erGO are ball milled together at various *B/C* weight ratios (0, ~ 0.09, ~ 0.36 and ~ 0.90) maintaining ~ 10 wt % of erGO in the nanocomposites. Several interactions among Mg, B and C, involving charge transfer, develop upon ball milling. Interestingly, a “critical” *B/C* ratio (~ 0.09) is identified. Below this ratio B is a charge (electron) donor to C. Above this ratio B accepts charge from Mg and also donates to C, acting as a net charge acceptor. When B is charge donor, a decrease in the incubation time is observed for H-uptake from ~ 3.25 h in the absence of B to ~ 2.25 h (i.e. a drop of ~ 31%) upon slight addition of B (*B/C* ≈ 0.09). Various fundamental scientific questions are answered: (i) How and why does B affect the Mg unit cell? (ii) How and why do interactions among Mg, B and C develop? (iii) Why does B switch the role from charge donor to net acceptor upon increasing its content? (iv) What is the impact of this role-switching by B on the H-uptake by the Mg-B-erGO nanocomposites? Our claims are supported by X-ray diffraction (XRD), X-ray photoelectron spectroscopy (XPS) (C-1s, Mg-2p and B-1s spectra) and the novel electron density maps, the technique which we used elsewhere^20^.

## Results

### Incubation time during H-uptake

**Figure 1:**
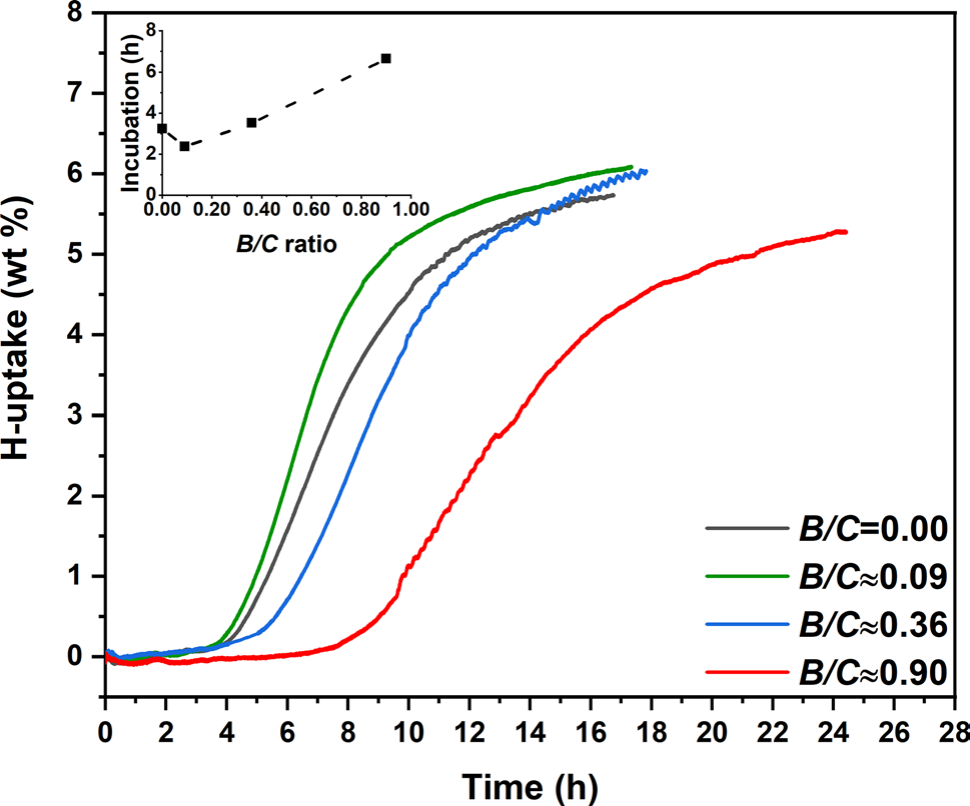
H-uptake (wt %) versus time (h) in ball milled Mg-B-erGO nanocomposites at various *B/C* ratios obtained at ~ 15 bar, ~ 320 °C. Inset shows the incubation time (h) plotted against *B/C* ratio.

The H-uptake by all the Mg-B-erGO nanocomposites at *P*_H2_ ≈ 15 bar and ~ 320 °C is plotted as wt % hydrogen in Mg versus time in Fig. 1. For all the nanocomposites, an incubation time corresponding to negligible H-uptake was seen before initiation of hydrogen absorption. These incubation times are plotted versus *B/C* ratio in the inset of Fig. 1. The incubation time decreases from ~ 3.25 h in *B/C* = 0 to ~ 2.25 h with a slight addition of B (*B/C*≈0.09). Surprisingly, with further addition of B the nanocomposites exhibit increased incubation time (inset, Fig. 1). This clearly indicates the existence of a critical *B/C* ratio around 0.09 at which the incubation time reaches a minimum. Moreover, following the incubation time all the H-uptake curves are sigmoidal in nature, prior to reaching saturation levels at ~ 6 wt %. The rate of H-uptake is the highest for *B/C*≈0.09. The nanocomposite with *B/C* ≈ 0.90 has the lowest rate of uptake and the longest incubation time. Figure 1 suggests that B influences the incubation time and the kinetics of H-uptake by these nanocomposites.

### Phase analysis

The X-ray diffraction (XRD) patterns of the ball milled nanocomposites are shown in Supplementary Fig. S1. The XRD pattern of the erGO is shown in Supplementary Fig. S2. The XRD patterns of the nanocomposites indicate the presence of hexagonal close packed (hcp) Mg. The crystallite size of Mg in all the ball milled nanocomposites is ~ 24 nm from Scherrer equation^21^. The patterns prior to background correction also show the possible presence of amorphous magnesium oxide along with the peaks corresponding to MgO, indicating oxidation of Mg^9^. One source of this oxygen could be from the functional groups present in erGO sheets, which could release while ball milling^22^. This oxygen could react with Mg and form oxide. Also, any possible mild exposure to air while transporting the nanocomposites could also have resulted in the formation of magnesium oxide. The phase percentages of Mg and MgO (crystalline) were estimated from XRD patterns through Rietveld refinement using FullProf suite (version: 7.20)^23^ and listed in Supplementary Table S1. The detailed procedure of Rietveld refinement is given in Supplementary Information. The fit curves, the residual fit errors are shown in Supplementary Fig. S3. The structure factors along with their uncertainties are reported in Supplementary Tables S2–S5.

### Structural changes in Mg unit cell

**Figure 2:**
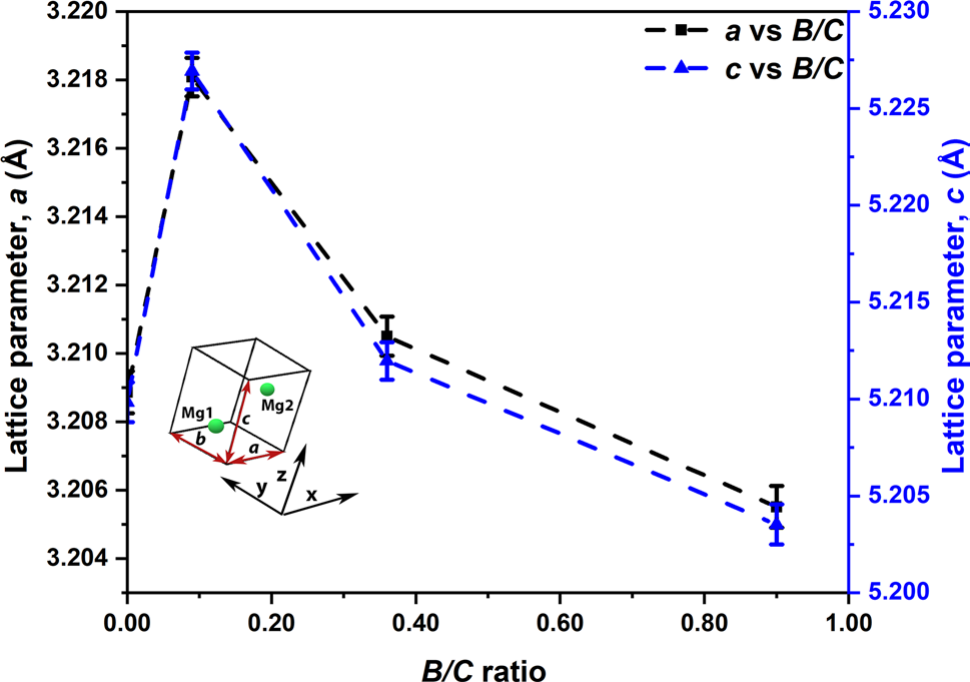
Lattice parameters of Mg (hcp) versus *B/C* ratio in ball milled Mg-B-erGO nanocomposites.

Figure 2 shows the lattice parameters of the Mg unit cell, estimated from XRD patterns through Rietveld refinement (see Supplementary Fig. S3 and Tables S2–S5) using FullProf suite (version: 7.20)^23^. Prior to analyzing the obtained lattice parameters, the structural integrity of the crystal for all the nanocomposites was verified and ensured by noting that the *c/a* ratio is close to the theoretical value of 1.623 for an hcp crystal^24^. Both *a* and *c* increase with an increase in the *B/C* ratio from 0 to ~ 0.09, indicating the Mg lattice expansion. These lattice parameters of *B/C* ≈ 0.09 are a real tendency, as suggested by the error bars and the structural integrity (*c/a* value) of the Mg unit cell at this composition. With further increase in *B/C* to ~ 0.36 and ~ 0.90 both *a* and *c* decrease, resulting in shrinkage of Mg lattice.

### Local environment in Mg unit cell

**Figure 3:**
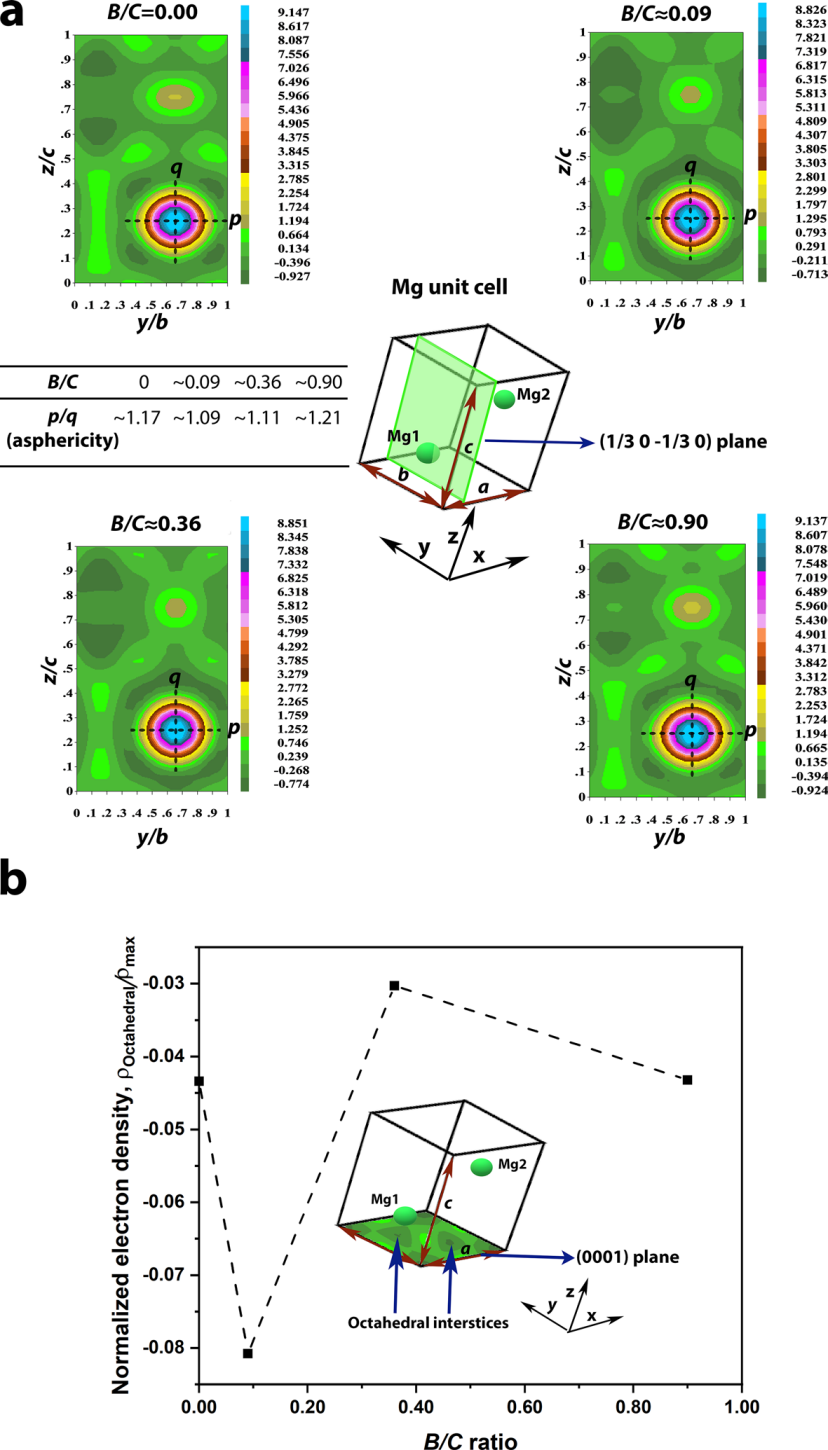
(**a**) Electron density maps corresponding to Mg (1/3 0 –1/3 0) plane and asphericity (*p/q*) of charge distribution around Mg1 atom at various *B/C* ratios; (**b**) Normalized electron density (*ρ*_Octahedral_*/ρ*_max_) at octahedral sites in Mg (0001) plane plotted against *B/C* ratio for ball milled Mg-B-erGO nanocomposites.

The local environment in the Mg unit cell is estimated by developing the electron density maps^25^ for the (1/3 0 –1/3 0) plane intersecting the atom ‘Mg1’ as shown in Fig. 3a. The colour-coded scales adjacent to the maps in Fig. 3a represent the electron density *ρ*(*r*) (= number of electrons/volume (Å^−3^)) values. It is to be noted that the positive value in these scales correspond to the presence of electrons and the negative values correspond to their absence. All the maps in Fig. 3a show that the maximum electron density is present in the regions, where the Mg atoms are located. This indicates the concentration of the maximum number of electrons around Mg. A careful observation of Fig. 3a suggests that the charge distribution around Mg is not similar in all the nanocomposites. This can be seen by the asphericity of the electron density distribution around Mg (*p/q*, Fig. 3a). The asphericity is estimated as the ratio of the distance along *b* to that along *c* from the center of Mg up to the outermost position, where the positive electron density can be observed. The estimated asphericities are ~ 1.17, ~ 1. 09, ~ 1.11 and ~ 1.21 for *B/C*s of 0, ~ 0.09, ~ 0.36 and ~ 0.90, respectively. Therefore, the electron density distribution is nearly uniform (asphericity closer to 1) for the nanocomposites of *B/C* ≈ 0.09. The aspherical charge distribution suggests covalency^20^. Figure 3b shows the relative electron density at the octahedral interstices in the (0001) basal plane of the Mg unit cell (‘Octahedral interstices’, Fig. 3b), normalized with respect to the maximum electron density found around Mg (i.e. *ρ*_Octahedral_*/ρ*_max_). The corresponding electron density maps are shown in Supplementary Fig. S4. It is known that the dopants can occupy these sites when incorporated in the Mg unit cell^26^. A negative value of this relative electron density indicates the absence of electrons. The relative electron densities of the nanocomposites with *B/C* of 0, ~ 0.36 and ~ 0.90 are negative, mutually comparable. The nanocomposite with *B/C* ≈ 0.09 has the least relative electron density; whereas, it is the highest for *B/C* ≈ 0.36. This suggests that among all the nanocomposites the octahedral sites in *B/C* ≈ 0.09 are least populated sites for electrons, suggesting a possible presence of a positively charged species.

### Chemical interactions

**Figure 4:**
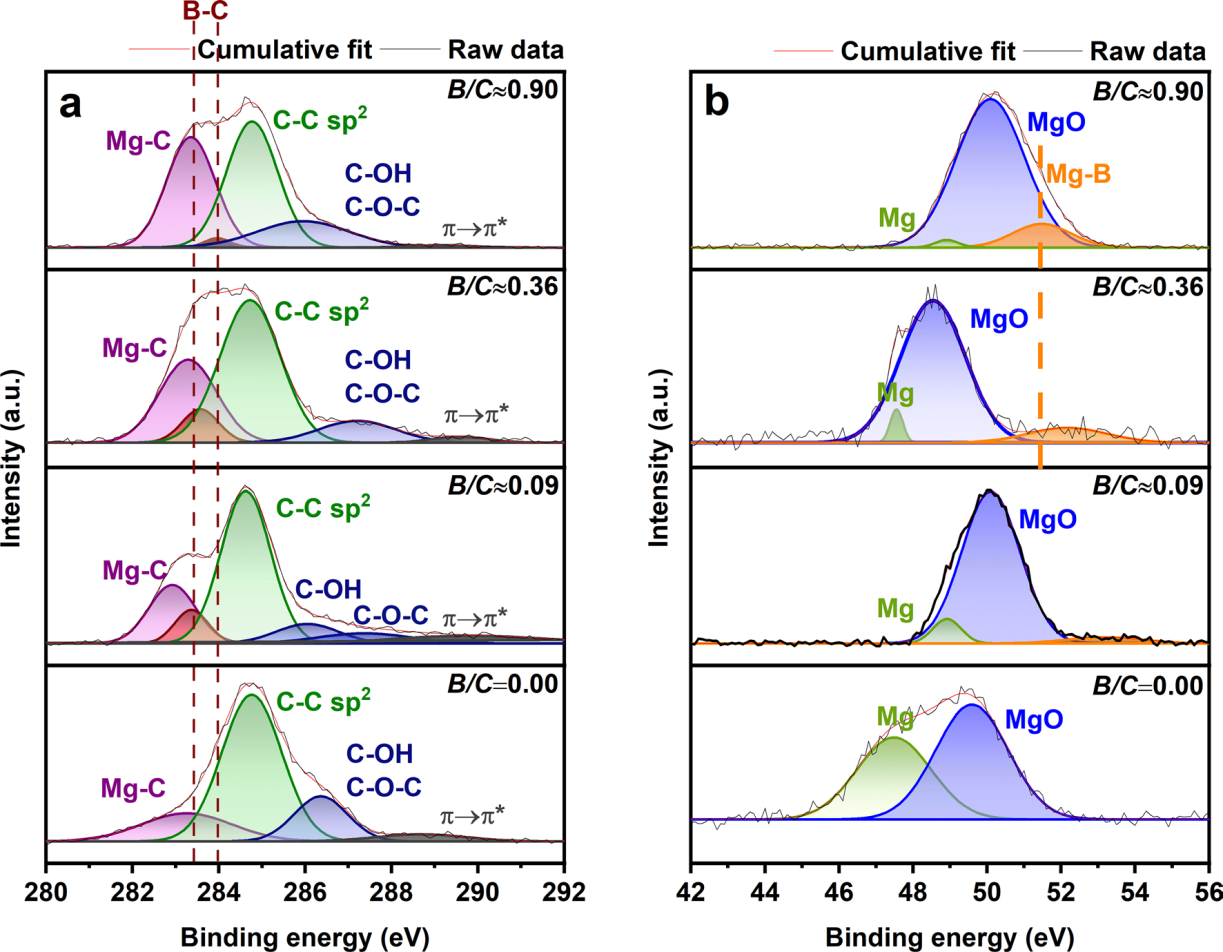
(**a**) C-1s core XPS spectra deconvoluted into Mg-C, B-C, sp^2^ hybridized C–C, C–OH, C–O–C and C-2p π → π* transition peaks; (**b**) Mg-2p spectra deconvoluted into metallic Mg, MgO and Mg-B for all the ball milled Mg-B-erGO nanocomposites.

The core C-1s and Mg-2p X-ray photoelectron spectra (XPS) of all the nanocomposites are shown in Fig. 4a,b, respectively. The experimental data obtained from XPS were deconvoluted using Gaussian profile by employing an XPS-specific deconvolution procedure to estimate the atomic interactions^27,28^. In C-1s spectra, the peaks at the binding energies ~ 283.2 eV, ~ 283.3–283.9 eV, ~ 284.6 eV, ~ 285.7 eV, ~ 286.6 eV, and ~ 290 eV correspond to Mg-C^18^, B-C^29,30^, C–C sp^2^, C–OH, C–O–C^31–33^ and C-2p π → π* transition^9,34^, respectively. The alcohol (–OH) and epoxy (C–O–C) functional groups arise during the synthesis of erGO (C-1s spectrum of erGO, Supplementary Fig. S5). The possible presence of COOH and C=O groups was also tested by considering them along with –OH and C–O–C groups in various combinations while deconvoluting the C-1s spectra. However, only those fits which contain only –OH and C–O–C groups were converged. The results of various such deconvolution exercises are presented in Supplementary Tables S6–S9. Moreover, as the erGO is synthesized using (NH_4_)_2_SO_4_ (see ‘Methods’ section), it is less likely for these groups to be present in erGO. Hence, it is believed that the COOH and C=O groups are absent in erGO. The XPS-specific deconvolution method^28^ resulted in acceptable FWHMs for both Mg-C and B-C peaks. For B-C peaks these FWHMs are 0.91, 1.00 and 0.70 eV for *B/C* ≈ 0.09, 0.36 and 0.90, respectively. In all the C-1s spectra the intensities of the Mg-C peaks are lower than those of the C–C sp^2^ peaks. From Fig. 4a, the Mg-C interactions in *B/C* = 0 are seen at ~ 283.2 eV. The deconvoluted spectrum of *B/C* ≈ 0.09 shows the B-C peak at ~ 283.4 eV^29^. With further increase in *B/C* to ~ 0.36 and ~ 0.90, the B-C and Mg-C peaks shifted towards higher binding energies. Interestingly, the C-1s spectrum of *B/C*≈0.90 possesses weaker B-C signal with lower FWHM as compared to that in the other spectra. The C-2p π → π* transition peak is visible in all the nanocomposites.

The peaks corresponding to metallic Mg and MgO are seen in all the compositions in Mg-2p spectra (Fig. 4b). The peaks of metallic Mg are expected within the binding energy range ~ 47.5–49.5 eV^35^. The charge of Mg in MgO (< + 2) causes an increase in binding energy, as the net number of electrons in the Mg valence shell in MgO are lower than those in metallic Mg^36^. The binding energy values of MgO in the present study lie within ~ 49–51 eV, consistent with literature^37^. The Mg-B peaks are seen in all the compositions and appear between ~ 51.3 and 53.2 eV^38,39^. With increase in B in the nanocomposites, the Mg-B peaks become stronger and shift towards lower binding energies in the above mentioned range (Fig. 4b). Interestingly, at *B/C*≈0.90 the binding energy of the Mg-B peak is around 51.3 eV. This is closer to the binding energy of Mg-B peak in MgB_2_^38^_._ The presence of both B-C (Fig. 4a) and Mg-B (Fig. 4b) peaks from XPS suggests that B has simultaneous interactions with both C and Mg.

## Discussion

The results suggest a critical *B/C* ratio around ~ 0.09, at which the incubation time during hydrogen uptake by the Mg-B-erGO nanocomposites is the least and increases above or below this ratio (Fig. 1). This critical *B/C* can be refined by selecting compositions around *B/C*≈0.09. The crystallographic (Fig. 2) and local environmental (Figs. 3 and 4) changes with the addition of B are used to understand the reasons behind the reduced incubation time. The presence of Mg, B and C renders the scenario complex by developing binary Mg-C, Mg-B, B-C and possible ternary Mg-B-C interactions upon ball milling. These interactions can affect the incubation time during H-uptake.

In the nanocomposite where B is absent (*B/C* = 0), the covalency of Mg as suggested by the aspherical charge distribution^40^ (*p/q*, Fig. 3a) indicates that there is an interaction between Mg and the surroundings. Further, the negative relative electron density in the octahedral interstices of the (0001) basal plane in Mg in *B/C* = 0 (Fig. 3b, Supplementary Fig. S4) shows the deficiency of electrons at this site. This suggests that there is no electron donation from Mg to this octahedral site. Therefore, it is reasonable to conclude that Mg donates electrons to C, which appears as ‘Mg-C’ peak in C-1s XPS spectrum (Fig. 4a). This electron donation from Mg to C can be reinforced by the fact that the electron affinity of Mg (~ 0 kJ mol^−1^) is lower than that of C (~ 153.9 kJ mol^−1^)^41^. Any local changes in the chemical environment can cause deviation from these values. In our earlier work, we demonstrated that the Mg-C interaction causes an increase of charge on carbon atoms leading to a change of hybridization in C from sp^2^ to sp^3^, resulting in the C-H bond aiding in H-uptake^18^.

A slight addition of B in *B/C* ≈ 0.09 decreases incubation time and enhances H-uptake kinetics (Fig. 1). This improvement is due to the structural and local environmental changes introduced by B. Upon addition of B, a significant expansion of the Mg unit cell can be seen from the lattice parameters in Fig. 2. Normally, the repulsive forces between two entities within a unit cell can cause its expansion^42^. In the present scenario, it can be expected that B is incorporated within the Mg unit cell and possesses a positive charge. The incorporation of B is suggested by the larger negative electron density compared to *B/C* = 0, i.e. lack of electrons, at the octahedral interstices in the (0001) Mg basal plane for *B/C*≈0.09 (Fig. 3b, Supplementary Fig. S4). In other words, the charge in these regions is more positive in *B/C*≈0.09 compared with that in *B/C* = 0. Most likely, B is present in these octahedral interstices^43^, while maintaining structural integrity, and possesses positive charge. The likely reason for the positive charge over B is the charge transfer from B to Mg and/or to C. However, the charge transfer from B to Mg is mostly not possible as the electron affinity of elemental B is ~ 28.9 kJ mol^−1^ and that of Mg is ~ 0 kJ mol^−1^^41^. Therefore, most likely, the charge transfer takes place from B to C, giving rise to B-C interaction as suggested by B-C peak at ~ 283.4 eV in C-1s spectrum (*B/C* ≈ 0.09, Fig. 4a). The absence of any peaks in B-1s core spectra for all the nanocomposites (Supplementary Fig. S6) shows that B substitution at C (~ 189.1 eV) and B_4_C (~ 187.7 eV), C_2_-BO (~ 191.4 eV), C-BO_2_ (~ 191.8 eV) are absent^44^. This is true despite the considerable concentrations of B in the nanocomposites (Supplementary Table S10). This clearly indicates that the B-C interactions seen in C-1s spectra (Fig. 4a) are not due to any strong bond formation between B and C. The presence of both Mg-C and B-C interactions in *B/C*≈0.09 show that both Mg and B are charge donors to C.

Upon increasing the B content in the nanocomposites to *B/C* ≈ 0.36 and 0.90 both the incubation time and the kinetics of H-uptake are deteriorated (Fig. 1). Interestingly, the lattice parameters in *B/C* ≈ 0.36 are close to those of *B/C* = 0 (Fig. 2) and have decreased further in *B/C* ≈ 0.90. The possible reasons for the restoration of the Mg unit cell to its original size are: (i) B is not present within the Mg unit cell at these compositions; (ii) B is present within the unit cell and shrinks its size to almost the original value by developing possible additional interactions. However, it is likely that B is incorporated in the Mg unit cell even at compositions higher than in *B/C* ≈ 0.09 (Fig. 3(b)). Therefore, the first reason is not plausible. Hence, the possible presence of B in the Mg unit cell is maintaining its size closer to its pristine counterpart (*B/C* = 0). From Fig. 3a, the charge distribution around Mg atom clearly shows that the asphericity increases (with respect to that at *B/C* ≈ 0.09) at *B/C* ≈ 0.36 and reaches the highest at *B/C* ≈ 0.90. This shows that the various interactions in *B/C* ≈ 0.36 and 0.90 are happening to a different extent compared with the other nanocomposites.

The different extents of the atomic interactions in *B/C* ≈ 0.36 and 0.90 are evident from the increase in the Mg-C and B-C binding energies with respect to those in *B/C* ≈ 0.09 from the C-1s spectra (Fig. 4a). The Mg-C and B-C peak positions increased from ∼ 282.9 and ∼ 283.4 eV (*B/C* ≈ 0.09) to ∼ 283.3 and ∼ 283.6 eV (*B/C* ≈ 0.36), respectively. Since the B-C peaks possess acceptable FWHMs (0.91 and 1.00 eV for B/C ≈ 0.09 and 0.36, respectively) these peak shifts in B-C are mostly realistic trends. This increase in the binding energies clearly indicates that a lower charge is received by C from both Mg and B^36^. An important question here is that: why does the charge reception by C decrease despite the charge donation by both Mg and B? The changes in the interactions of C with Mg and B also introduce observable interactions between Mg and B in *B/C* ≈ 0.36. The Mg-2p spectrum for this composition shows the presence of Mg-B peak at ~ 52.2 eV, corresponding to the charge transfer from Mg to B^38^ (Fig. 4b). This Mg-B peak in *B/C* ≈ 0.09 is very feeble, rendering the relative charge transfer from Mg to B^38^ in *B/C* ≈ 0.36 very significant. The combined analysis of C-1s and Mg-2p spectra (Fig. 4) clearly indicates that Mg is donating charge to both C and B. As a result of this, the net charge received by C from Mg can decrease (Fig. 4a). Similarly, as B shares its valence electrons with Mg, more likely a lower net charge is received by C from B (Fig. 4a). The presence of both Mg-B and B-C interactions can render B a charge acceptor (from Mg) and a donor (to C), respectively. However, the relative electron richness at octahedral sites (*B/C* ≈ 0.36, Fig. 3b), which B is likely occupying, suggests that B is negatively charged making it a net charge acceptor. The results indicate that ternary Mg-B-C interactions develop in *B/C* ≈ 0.36. Similar trends in the interactions are observed in the case of *B/C* ≈ 0.90 (Fig. 4).

The nanocomposite with *B/C* ≈ 0.90 exhibits asphericity close to that of *B/C* = 0 (Fig. 3a). Its *ρ*_Octahedral_*/ρ*_max_ value is negative and similar to that of *B/C* = 0 (Fig. 3b, Supplementary Fig. S4). Despite these similarities, it shows longer incubation time (Fig. 1) for H-uptake. This suggests that, possibly, further different level of interactions are present in this nanocomposite, as implied by the lowest unit cell volume in this case (Fig. 2). From Fig. 4a, the Mg-C and B-C peaks for *B/C* of 0 (only Mg-C), ∼ 0.09 and ∼ 0.36 are shifted to lower binding energies compared with those in *B/C* ≈ 0.90 suggesting stronger interactions. The Mg-B peak is located at ~ 51.3 eV (Fig. 4b). Interestingly, this is the position where the similar peak in MgB_2_ is expected^45^. Moreover, B is expected to be located in the octahedral interstices in Mg unit cell (hcp) in *B/C* ≈ 0.90 (Fig. 3a), which are the cites where B is located in MgB_2_ (also hcp)^46^. However, the XRD pattern (Supplementary Fig. S1) does not show any MgB_2_ peaks [ICSD code: 26675]. Probably, the composition *B/C* ≈ 0.90 is just about favorable for the formation of MgB_2_. The possible presence of MgB_2_ in *B/C*≈0.90 is also supported by the lowest unit cell volume (Fig. 2), the highest asphericity/covalency (Fig. 3a), the presence of the highest Mg-C and B-C binding energies (Fig. 4a) and a peak corresponding to MgB_2_ (51.3 eV) in Mg-2p spectra (Fig. 4b). However, additional characterization is needed to substantiate the presence of MgB_2_ in this nanocomposite.

**Figure 5:**
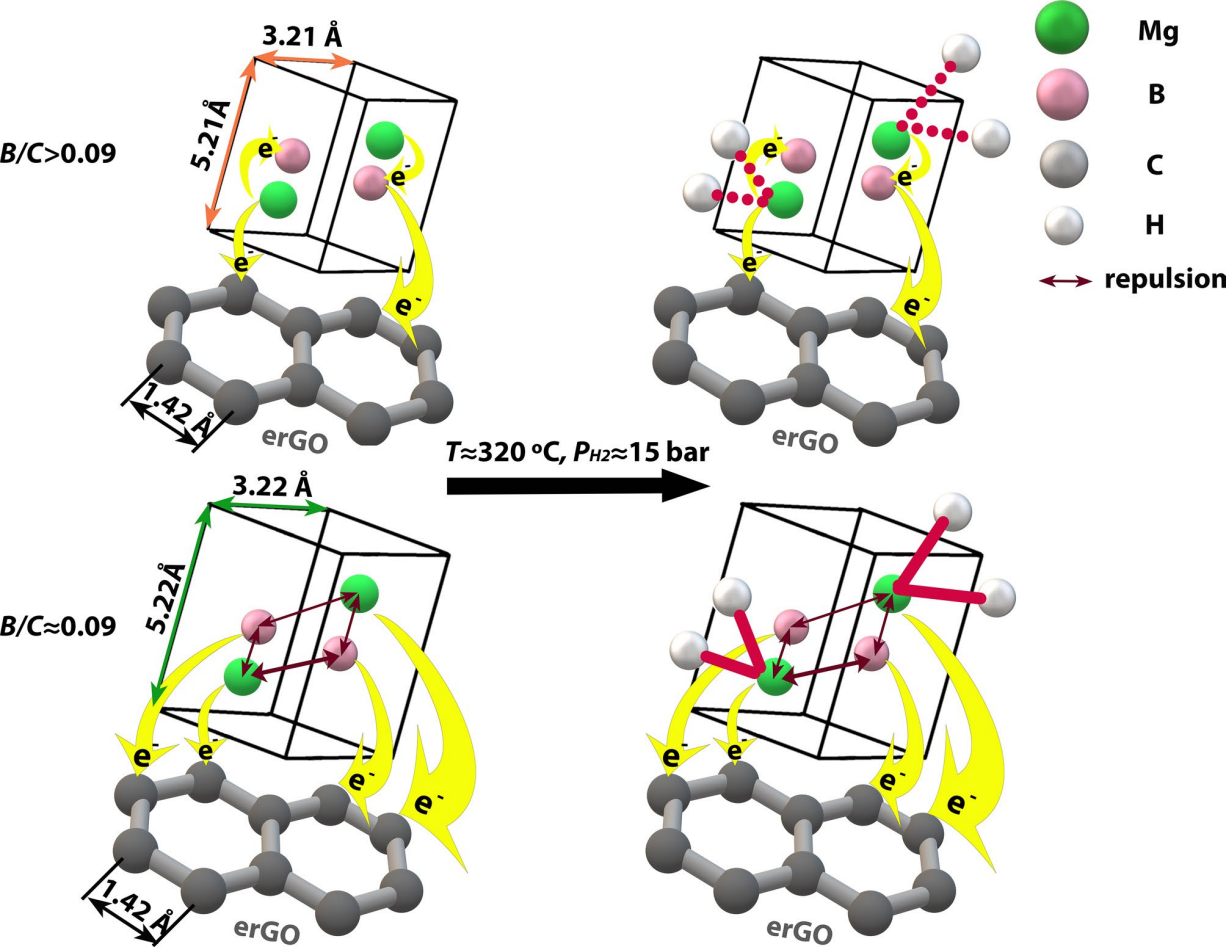
Various interactions among Mg, B, C at *B/C* > 0.09 and ~ 0.09 in Mg-B-erGO nanocomposites after ball milling and while H-uptake.

The analysis from the present study is used to propose a mechanism in terms of the structural and local environmental changes in the Mg-B-erGO nanocomposites that help in reducing the incubation time during their H-uptake. This mechanism is schematically shown in Fig. 5. Among the investigated compositions, those at *B/C* > 0.09 develop ternary Mg-B-C atomic interactions, where C from erGO receives charge from both Mg and B (Mg-C, B-C in Fig. 4a). Interestingly, B also receives charge from Mg developing Mg-B interactions (Fig. 4b). Such a charge reception renders B negative making it a net charge acceptor. This is evidenced by the**,** relatively, electron-rich octahedral interstices in Mg unit cell, where there is a likelihood of B’s presence (Fig. 3b, Supplementary Fig. S4). The attraction between the negatively charged B and Mg helps in maintaining the Mg unit cell size in *B/C*≈0.36 almost the same as that in *B/C* = 0 (Fig. 2). Despite the charge donation to both C and B, the charge on Mg is <  + 2 rendering it difficult to bond with H (dotted line between H and Mg, Fig. 5). This results in the longer incubation times at *B/C* > 0.09 (Fig. 1). In the case of *B/C* ≈ 0.90 the longest incubation time can be attributed to the possible presence of MgB_2_. However, this needs to be confirmed with further analysis.

At *B/C* ≈ 0.09, B acts as a charge donor to C (B-C, Fig. 4a). The possible presence of the positively charged B in the octahedral interstices of Mg unit cell (*ρ*_Octahedral_*/ρ*_max_, Fig. 3b, Supplementary Fig. S4) repels Mg (‘repulsion’, Fig. 5) and causes the lattice expansion (Fig. 2). Here, Mg is more positive relative to that at *B/C* > 0.09 due to the lower binding energy of Mg-C (Fig. 4a) rendering Mg-H bond stronger (solid line between H and Mg, Fig. 5) than in *B/C* > 0.09. The more positive Mg can bond strongly with H and can reduce the incubation time (Fig. 1)^18^.

The present study shows that various interactions among Mg, B and C in the Mg-B-erGO nanocomposites influence the reduction of incubation time and increase in the H-uptake kinetics in the order: (Mg → C; B → C)_*B/C*≈0.09, *B*: donor_ > (Mg → C)_*B/C*=0_ > (ternary Mg → B → C)_*B/C*>0.09, *B*: acceptor_.

## Methods

Electrochemically reduced graphene oxide (erGO) was synthesized by electrochemical exfoliation of graphite rod (*Φ*: 305 mm, CAS: 40765, Alfa Aesar) using CHI 660E electrochemical work station in 0.1 M (NH_4_)_2_SO_4_ aqueous electrolyte. Platinum mesh and Ag/AgCl (3 M KCl) were used as counter and reference electrodes, respectively. Potentiostatic exfoliation at 3 V for 3 h resulted in exfoliation of graphene sheets from the surface of graphite rod because of (SO_4_)^2-^ ion intercalation^47^.

Magnesium (Sigma Aldrich; ~ 44 µm; > 99%), amorphous boron powder (Sigma Aldrich; ≤ 1 µm; > 95%) were used along with erGO for synthesizing Mg-B-erGO nanocomposites. Four different nanocomposites with *B/C* weight ratios of 0, ~ 0.09, ~ 0.36 and ~ 0.90 were synthesized. The corresponding *B/Mg* weight ratios are 0, ~ 0.01, ~ 0.04 and ~ 0.10, respectively. The erGO is ~ 10 wt % with respect to the total weight of the nanocomposite. All the samples in the present study are referred with respect to their *B/C* weight ratios to understand the synergetic effect of the catalysts B and C on H-uptake by Mg. All the materials were handled in argon atmosphere using MBraun Unilab Plus 4-port glovebox maintaining oxygen (O_2_) and moisture (H_2_O) levels at < 0.1 ppm. The Mg-B-erGO nanocomposites were synthesized through ball milling at 450 rpm for 20 h by loading Mg, B and erGO in appropriate compositions into a 45 ml tungsten carbide (WC) vessel containing WC balls maintaining a ball to powder weight ratio of 40:1. A Fritsch pulverisette 7, premium line planetary micro mill was employed for ball milling.

Following ball milling the nanocomposites were loaded and sealed in a hydrogenation reactor inside glovebox. Subsequently, the reactor was brought out for H-uptake experiments in a Sievert’s type apparatus. Prior to H-uptake, the reactor was purged with Ar. The reactor containing the powder was heated up to ~ 320 °C under vacuum. Eventually, hydrogen gas (99.999%) was permitted in to the reactor at ~ 15 bar and ~ 320 °C. Isothermal H-uptake experiments were conducted on these Mg-B-erGO nanocomposites at these conditions up to saturation. The quantity of H-uptake by the powders was estimated using ideal gas law^20^.

The phase analysis on these nanocomposite powders was performed by X-ray diffraction (XRD) employing PANalytical EMPYREAN goniometer. Cu Kα radiation (wave length: 1.5406 Å) was used for the same. The baseline correction was performed on the obtained data and the phases were indexed using the standard ICSD references. The ICSD database codes used for indexing Mg, MgO and rGO phases are 76748, 104845 and 31170, respectively.

The XRD patterns of the nanocomposites were subjected to Rietveld refinement using FullProf suite (version: 7.20) to estimate phase percentages and to obtain crystallographic data of phases^23^ shown in Supplementary Fig. S3 and Tables S2–S5. Baseline for the experimentally obtained data was corrected using winPLOTR program^48^. After baseline correction, the data was refined using Pseudo-Voigt function^49^ (Supplementary Eq. S8). The lattice parameters (*a, b, c, α, β, γ*) of hcp Mg, FWHM (*U, V, W, IG*), shape (*η*_*0,*_* X*) and asymmetry were refined to obtain the best fit with the experimental data for Mg and MgO phases (Supplementary Eqs. S4, S9). For refining the lattice parameters, the initial values of *a* = 3.2093 Å, *b* = 3.2093 Å and *c* = 5.2103 Å were fed as input into EdPCR application of the FullProf suite prior to Rietveld refinement. The electron density maps are used to estimate the local environment within the crystal lattice. Electron density maps for Mg unit cell were developed using GFourier Program (version: 4.06) through Maximum Entropy Method (MEM)^25^. Through MEM the electron density *ρ*(*r*) was calculated by Fourier transformation of structure factors obtained after Rietveld refinement^50^ (Supplementary Eq. S11).

X-ray photoelectron spectroscopy (XPS) was performed on the nanocomposites employing Axis Supra Photoelectron spectrometer (Kratos Analytical) using Al Kα source. The powders were ultrasonicated in toluene for uniform dispersion following which they were drop casted onto Al foil and exposed to XPS source maintaining 20 eV pass energy. The obtained high resolution XPS spectra for various orbitals were deconvoluted using Gaussian function employing an XPS-specific deconvolution method for estimating the chemical bonds^28^.

## Supplementary Information

Supplementary Information.
