# Supplementary material for: Boron from net charge acceptor to donor and its effect on hydrogen uptake by novel Mg-B-electrochemically synthesized reduced graphene oxide
Source: Sci Rep. 2021 May 26;11:10995. doi: 10.1038/s41598-021-90531-w (PMC8154900; doi:10.1038/s41598-021-90531-w)
Supplement: Supplementary file 1 — Supplementary Information. [file 41598_2021_90531_MOESM1_ESM.pdf]

Supplementary Information

Boron from net charge acceptor to donor and its effect on hydrogen uptake by novel Mg-B-electrochemically synthesized reduced graphene oxide

Marla V V Satya Aditya, Srikanta Panda, and Sankara Sarma V Tatiparti*

Department of Energy Science & Engineering, Indian Institute of Technology Bombay, Mumbai 400076, India

***Corresponding author E-mail: sankara@iitb.ac.in**

S1. X-ray diffraction (XRD)

XRD patterns of magnesium (Mg)-boron (B)-electrochemically synthesized reduced graphene oxide (erGO) nanocomposites are shown in Fig. S1. The peaks at ~32.2°, ~34.4°, ~36.6°, ~47.8°, ~57.4°, ~63.1°, ~67.3°, ~68.6°, ~70.2°, ~72.5°, ~77.8° and ~81.5° correspond to hexagonal close packed structure (hcp) of Mg phase [ICSD code: 76748]. The minor peak at ~42.8° corresponds to MgO [ICSD code: 104845]. Peaks corresponding to erGO are not observed.

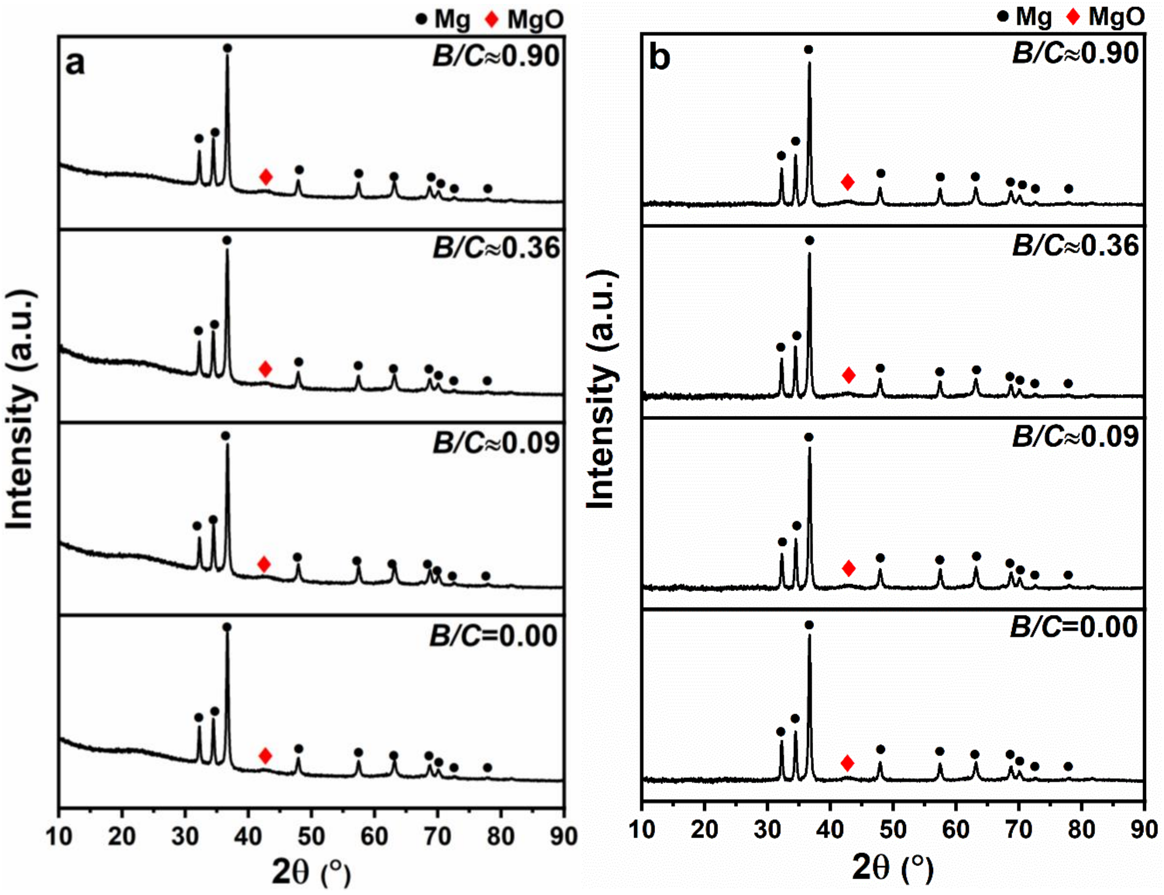

Figure S1. XRD patterns (a) without background correction, and (b) with background correction; for ball milled Mg-B-erGO nanocomposites with various *B/C* ratios.

XRD pattern of erGO is shown in Fig. S2. Peaks corresponding to ~26.56°, ~42.39°, ~44.56°, ~54.48°, and ~77.61° correspond to carbon [ICSD code: 31170].

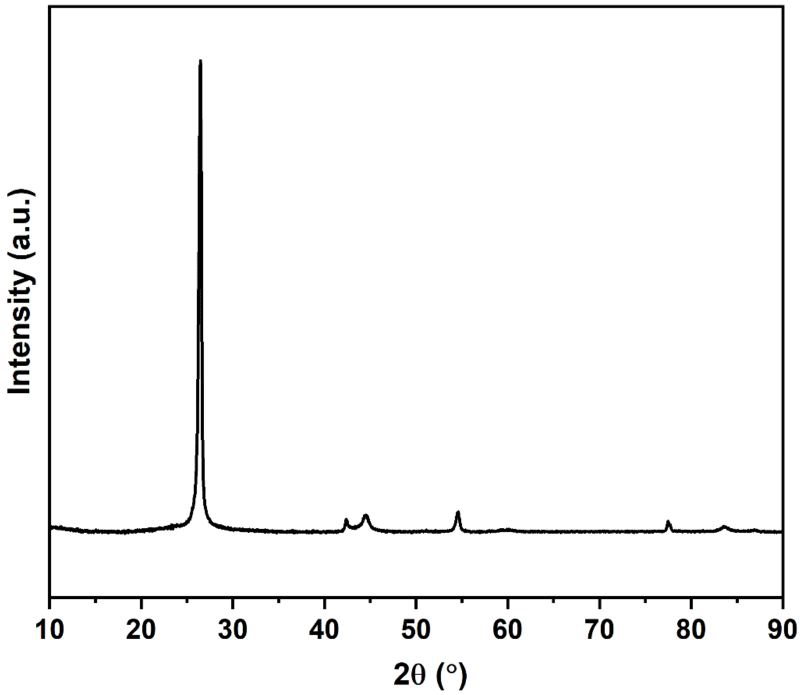

Figure S2. XRD pattern for electrochemical reduced graphene oxide (erGO).

S2. Rietveld refinement

The data obtained from XRD technique can yield significant crystallographic information such as lattice parameters, structure factors etc., of the individual phases along with their respective phase percentages present within the sample. To obtain such information, "Rietveld refinement" is used to refine the XRD data[1]. Prior knowledge of the crystallographic information viz. space groups of the individual phases that are likely to be present within the sample is necessary for the refinement. In the present case, Mg and MgO phases are present within the sample as seen from Supplementary Fig. S1. Also, Magnesium boride (MgB_2) can form in these Mg-B-erGO nanocomposites.

A commercial software, FullProf suite (version: 7.20) that is widely used for Rietveld refinement is employed[2]. Initially, the background (Supplementary Fig. S1a) of the raw XRD patterns was corrected using winPLOTR program[3]. Instrumental factors or likely presence of any non-stoichiometric compound (amorphous MgO) lead to this background. The parameters needed for initiating the refinement viz. individual space groups, peak positions and lattice parameters are obtained from the standard ICSD reference data for the phases Mg [ICSD code: 76748], MgO [ICSD code: 104845]and MgB_2 [ICSD code: 93925].

The peaks seen in XRD pattern generally follow a mathematical function, Pseudo-Voigt ($pV(x)$)[4]. Pseudo-Voigt is a linear combination of Gaussian ($G(x)$, Eq. (S1)) and Lorentzian ($L(x)$, Eq. (S5)) functions. All the other parameters used in these Gaussian and Lorentzian functions are listed in the Eqs. S2-S4, S6 and S7. The Pseudo-Voigt ($pV(x)$) function is given in Eq. S8. The weighted fraction of η used for combining the Gaussian and Lorentzian functions to obtain the Pseudo-Voigt function is given in Eq. S8.

Gaussian function $\left(G(x)\right)$:

$$G(x)|_{x=-1\ to\ 1} = a_g\ exp\left(-b_g x^2\right) \quad \text{(S1)}$$

where,

$$a_G = \frac{2}{FWHM}\sqrt{\frac{ln\ 2}{FWHM^2}} \quad \text{(S2)}$$

$$b_G = \frac{4\ ln2}{FWHM^2} \quad \text{(S3)}$$

FWHM: Full width half maximum

$$FWHM^2 = \left(U + {D_{ST}}^2\right)(\tan\theta)^2 + V\tan\theta + W + \frac{I_G}{(\cos\theta)^2} \quad \text{(S4)}$$

where, U, V, W and I_G are FWHM parameters

Lorentzian function $\left(L(x)\right)$:

$$L(x)|_{x=-1\ to\ 1} = \frac{a_L}{1+b_L x^2} \quad \text{(S5)}$$

where,

$$a_L = \frac{2}{\pi FWHM} \quad \text{(S6)}$$

$$b_L = \frac{4}{FWHM^2} \quad \text{(S7)}$$

Pseudo-Voigt function $\left(pV(x)\right)$:

$$pV(x) = \eta\, L(x) + (1-\eta)G(x) \quad \text{(S8)}$$

where,

$$\eta = \eta_0 + X2\theta \quad \text{(S9)}$$

where, η_0 and X are shape parameters

The values U, V, W, η_0 and X are varied up to a point where a minimum χ^2 fit is achieved for the refined data. The patterns corresponding to the fitted, experimental patterns and the residual errors obtained are shown in Fig. S3.

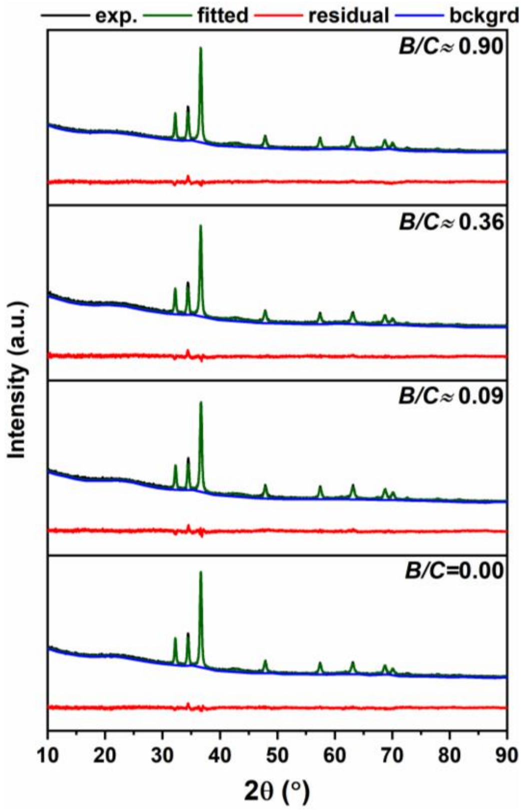

Figure S3. Fitted XRD patterns after Rietveld refinement for ball milled Mg-B-erGO nanocomposites with various *B/C* ratios.

S2.1 Phase percentages

The phase percentages of Mg and MgO were estimated from Rietveld refinement (shown in Table S1). It is noticed that the refinement did not yield any presence of MgB_2 in the nanocomposites. This suggests that any likely presence of MgB_2 is below the detection limits of XRD.

Table S1. Phase percentages of Mg and MgO in the ball milled Mg-B-erGO nanocomposites with various *B/C* ratios.

***B/C* ratio**	**Mg (%)**	**MgO (%)**	**Convergence (χ^2)**
0.00	89.21	10.79	2.42
0.09	81.60	18.40	2.98
0.36	78.22	21.78	2.27
0.90	86.01	13.99	2.82

S2.3. Structure factors

The structure factors (*F*) of the planes are estimated from the intensity (*I*) of the XRD peaks obtained after refinement. The relation between intensity (*I*) and structure factors (*F*) is as given in Eq. S10[5].

$$I \propto |F|^2 \quad \text{(S10)}$$

The structure factor (*F*) is Fourier transform that possesses a unique value at each (*hkl*) reflection and follows the relation shown in Eq. S11[5].

$$F(hkl) = \sum_h \sum_k \sum_l f_{Mg}.exp(2\pi i \alpha_{hkl}) \quad \text{(S11)}$$

where, f_{Mg}: atomic scattering factor of Mg atom and α_{hkl} is given in Eq. S(12).

$$\alpha_{hkl} = h.u + k.v + l.w \quad \text{(S12)}$$

where, $u = \frac{x}{a}; v = \frac{y}{b}; w = \frac{z}{c}$

Here, (*x*, *y*, *z*) is the position of Mg atom; *a*, *b* and *c* are the lattice parameters of Mg unit cell.

The estimated structure factors and their uncertainties (σ) (estimated from the standard deviations in *a*, *b* and *c*) for all the *B/C*s are shown in Tables S2-S5

Table S2. Structure factors and their uncertainties for Mg phase at *B/C*≈0.00

2θ (◦)	h	k	l	$\|F\|$	$\sigma\|F\|$
32.184	1	0	0	9.178	0.003551384
34.399	0	0	2	18.012	0.000004284
36.619	1	0	1	15.316	0.010051108
47.820	1	0	2	8.088	0.012792928
57.383	1	1	0	14.949	0.000003358
63.073	1	0	3	12.331	0.017904565
67.334	2	0	0	6.861	0.005311566
68.645	1	1	2	13.566	0.000012539
70.023	2	0	1	11.608	0.010607509
72.513	0	0	4	13.116	0.000012478
77.849	2	0	2	6.261	0.012330106
81.557	1	0	4	6.065	0.016836811

Table S3. Structure factors and their uncertainties for Mg phase at *B/C*≈0.09

2θ (◦)	h	k	l	$\|F\|$	$\sigma\|F\|$
32.092	1	0	0	9.186	0.003231035
34.313	0	0	2	18.030	0.000003642
36.516	1	0	1	15.330	0.009214777
47.688	1	0	2	8.098	0.011754257
57.208	1	1	0	14.972	0.000002779
62.895	1	0	3	12.355	0.016476042
67.122	2	0	0	6.874	0.004837472
68.435	1	1	2	13.593	0.000010516
69.802	2	0	1	11.632	0.009715331
72.309	0	0	4	13.147	0.000010597
77.599	2	0	2	6.276	0.011320347
81.311	1	0	4	6.082	0.015512530

Table S4. Structure factors and their uncertainties for Mg phase at *B/C*≈0.36

2θ (◦)	*h*	*k*	*l*	|*F*|	*σ*|*F*|
32.170	1	0	0	9.179	0.002960276
34.386	0	0	2	18.014	0.000003042
36.603	1	0	1	15.318	0.008433722
47.799	1	0	2	8.090	0.010752178
57.356	1	1	0	14.953	0.000002333
63.044	1	0	3	12.334	0.015061684
67.301	2	0	0	6.863	0.004428066
68.613	1	1	2	13.570	0.000008811
69.988	2	0	1	11.612	0.008886475
72.481	0	0	4	13.120	0.000008861
77.811	2	0	2	6.264	0.010348062
81.518	1	0	4	6.068	0.014170007

Table S5. Structure factors and their uncertainties for Mg phase at *B/C*≈0.90

2θ (◦)	*h*	*k*	*l*	|*F*|	*σ*|*F*|
32.174	1	0	0	9.175	0.003363821
34.395	0	0	2	18.006	0.000003909
36.608	1	0	1	15.311	0.009568200
47.809	1	0	2	8.085	0.012191784
57.364	1	1	0	14.941	0.000003013
63.059	1	0	3	12.323	0.017069026
67.311	2	0	0	6.856	0.005028963
68.625	1	1	2	13.556	0.000011344
69.999	2	0	1	11.600	0.010079677
72.502	0	0	4	13.104	0.000113793
77.825	2	0	2	6.256	0.011731393
81.541	1	0	4	6.059	0.016051430

S3. Electron density maps

The electron density maps are used to estimate the local environment within the crystal lattice. Electron density maps for Mg unit cell were developed using GFourier Program (version: 4.06) through Maximum Entropy Method (MEM)[6]. The electron density $\rho(r)$ (no. of electrons per unit volume (Å^{-3})) was calculated by Fourier transformation of structure factors obtained as shown in the below equation[7]:

$$\rho(r) = \frac{1}{V}\sum_H F(H)exp\{-2\pi i(H(r))\} \qquad \text{(S11)}$$

Where, V is volume of the unit cell; H is a reciprocal lattice vector, r is a vector position inside the unit cell; $F(H)$ is a Fourier transform of the structure factor.

The electron density maps developed from octahedral interstices of Mg unit cell are shown in the below Fig. S4.

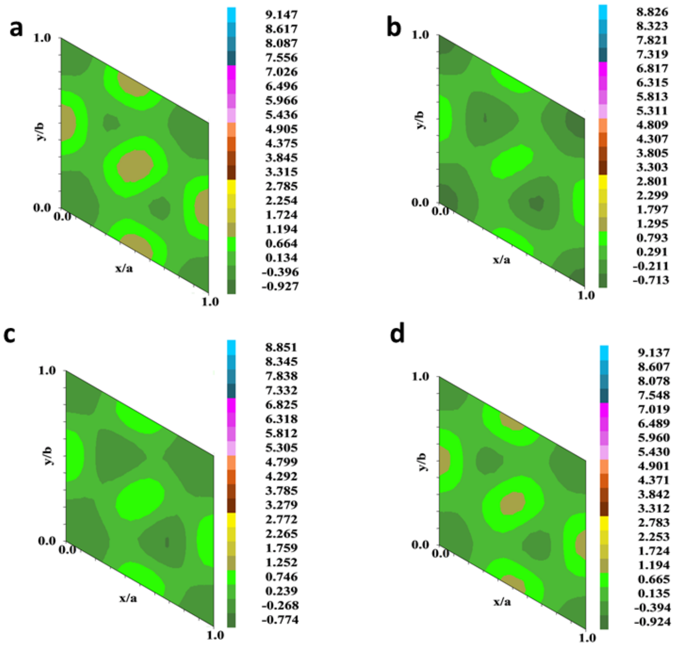

Figure S4. Electron density maps corresponding to (0001) plane of Mg unit cell for ball milled Mg-B-erGO nanocomposites with various *B/C* ratios.

S4. X-ray photoelectron spectroscopy (XPS) and its deconvolution

When the true XPS spectrum is separated from the observed spectrum, the details of the chemical environment can be revealed. This involves eliminating broadening of the spectra caused by the instrumental factors. The spectra are therefore deconvoluted by a careful consideration of the following factors[8]:

1.) Choosing an appropriate background to filter noise and obtain desirable ratios of fit components.
2.) The line widths of the deconvoluted peaks lie within an acceptable limit to prevent overlapping of peaks.
3.) The line widths of the deconvoluted peaks are above an acceptable limit to convey the physical significance of a chemical bonds existing within the sample.

In the present Mg-B-erGO nanocomposites, the peaks corresponding to Mg-C, B-C interactions, C-C sp^2 peak, C-OH, C-O-C peaks and $\pi \rightarrow \pi^*$ transitions were obtained after deconvoluting C-1s spectra. However, only a single peak corresponding to oxygen (O) containing functional groups (viz. C-OH, C-O-C, C=O, COOH etc.) was obtained. The attempts made to achieve optimum peaks (FWHMs, intensities and peak positions) that reveal the extent of O-containing functional groups are listed in Tables S6-S9 for various *B/C* ratios.

Table S6. Various trends of the cumulative fits obtained while fitting C-1s spectrum of *B/C*=0.00.

**Functional groups**	**R^2**	**Remarks**	**Plot**
C-OH and π⟶π*	99.71	π⟶π* transition peak shifted to 288.62 eV	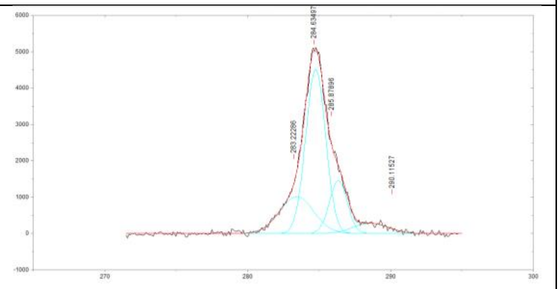
C-O-C and π⟶π*	99.71	π⟶π* transition peak shifted to 288.62 eV	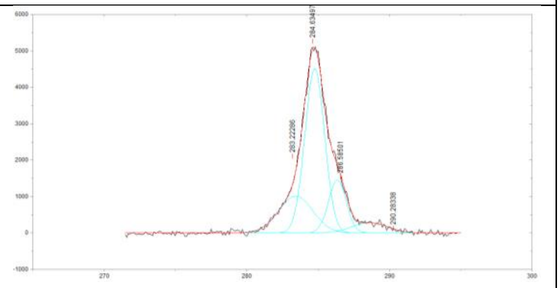
C=O and π⟶π*	Not converged	A negative peak.<br>Large peak lying outside the raw spectrum	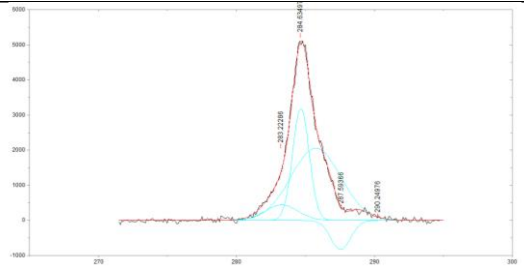
COOH and π⟶π*	Not converged	A negative peak.<br>Large peak lying outside the raw spectrum	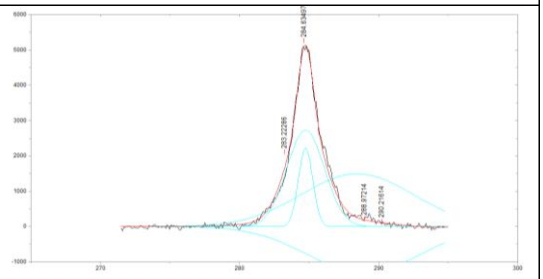
C-OH, COOH and π⟶π*	Not converged	A large negative peak<br>Large peak lying outside the raw spectrum	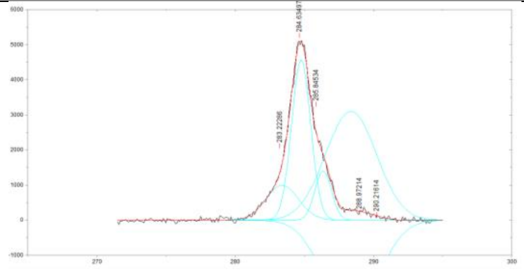

C-OH, C=O and π⟶π*	Not converged	Infinite FWHM at 286.35 eV	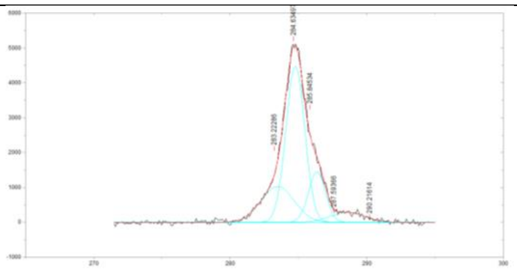
C-OH, C-O-C and π⟶π*	Not converged	Negative peak at 286.36	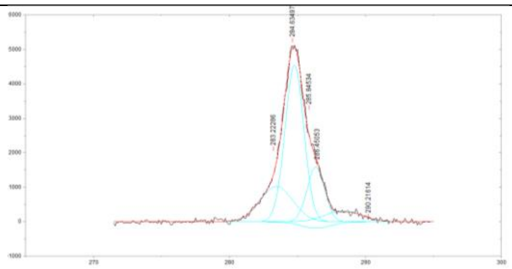
C-O-C, COOH and π⟶π*	Not converged	Infinite FWHM at 286.35 eV	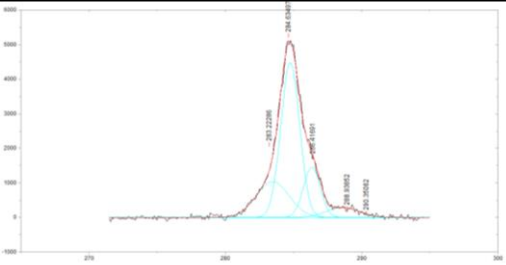
C-O-C, C=O and π⟶π*	Not converged	Infinite shift in B.E and infinitely large FWHM	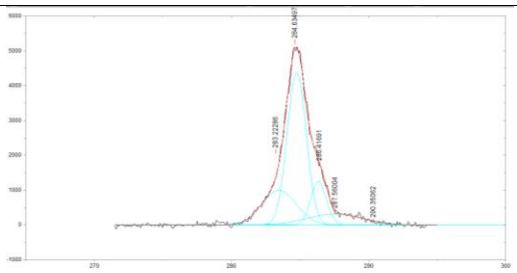
C=O, COOH and π⟶π*	Not converged	Large negative peak<br>Two peaks lying outside the raw spectrum	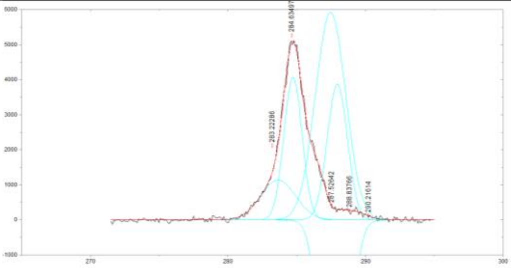
C-OH, C-O-C, C=O and π⟶π*	Not converged	Negative peak at 290.14 eV	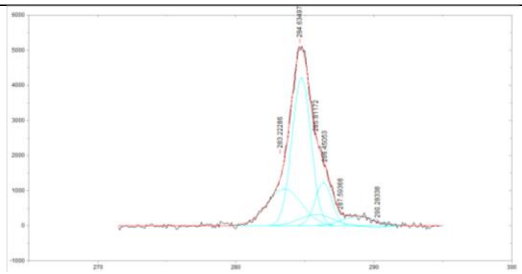

C-OH, C-O-C, COOH and π⟶π*	Not converged	Two peaks at same B.E (284.76 eV)<br><br>A negative peak at 290.16 eV	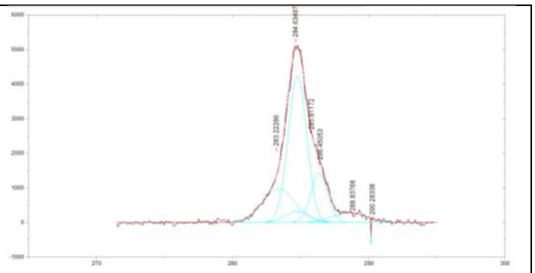
C-OH, C=O, COOH and π⟶π*	Not converged	Negative peak of infinite intensity<br><br>Large FWHM peak at 286.25 eV	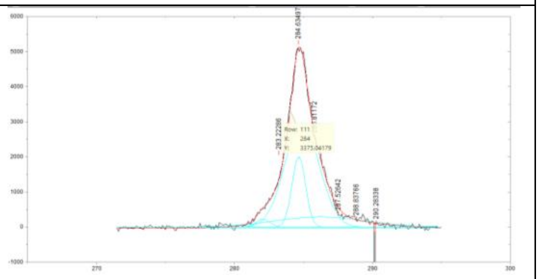
C-O-C, C=O, COOH and π⟶π*	Not converged	Negative peak of infinite intensity at 282.05 eV<br><br>Two peaks at same B.E. (284.76 eV)	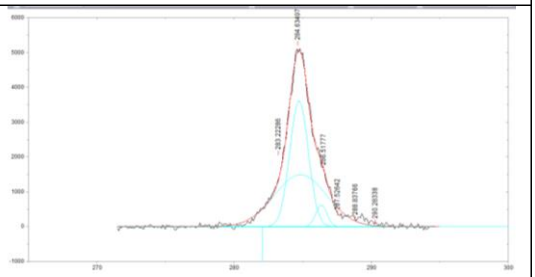
C-OH, C-O-C, C=O, COOH and π⟶π*	Not converged	Negative peak at 287.55 eV<br><br>Negative peak at 290.11 eV with infinite intensity	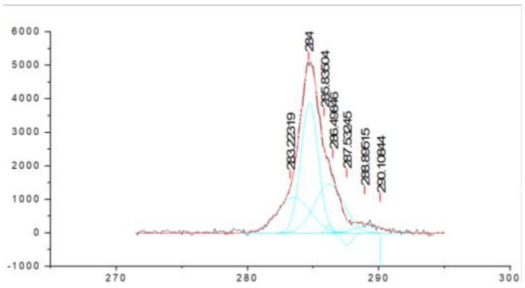

Table S7. Various trends of the cumulative fits obtained while fitting C-1s spectrum of *B/C*≈0.09.

Functional groups	R^2	Remarks	Plot
C-OH and $\pi \rightarrow \pi^*$	99.81	Broad peak at 288.08 eV<br>No $\pi \rightarrow \pi^*$ transition peak	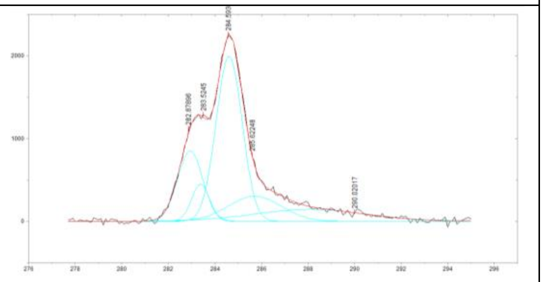
C-O-C and $\pi \rightarrow \pi^*$	99.81	Broad peak at 288.08 eV	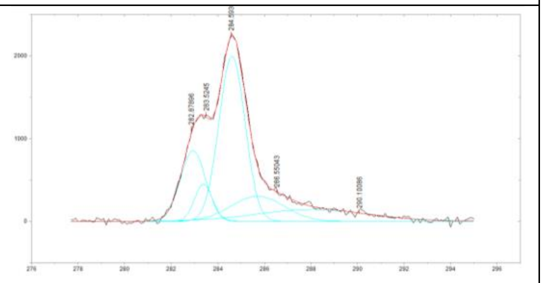
C=O and $\pi \rightarrow \pi^*$	Not converged	A broad peak at 286.16 eV<br>A peak's intensity lying outside the spectrum at 290.16 eV	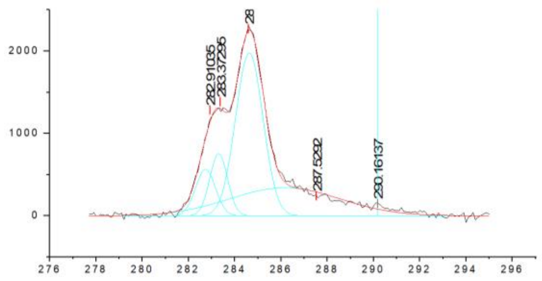
COOH and $\pi \rightarrow \pi^*$	Not converged	An infinitely large peak at 287.01 eV<br>An infinitely large negative peak at ~287.25 eV	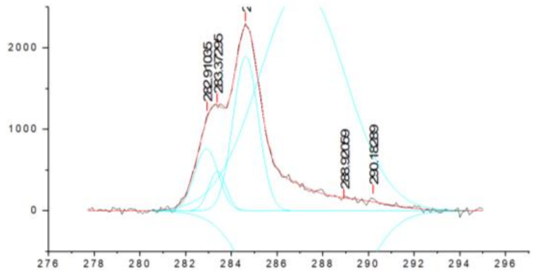
C-OH, C-O-C and $\pi \rightarrow \pi^*$	99.81	Two peaks at same B.E (286.17 eV)<br>A broad peak at 289.6 eV	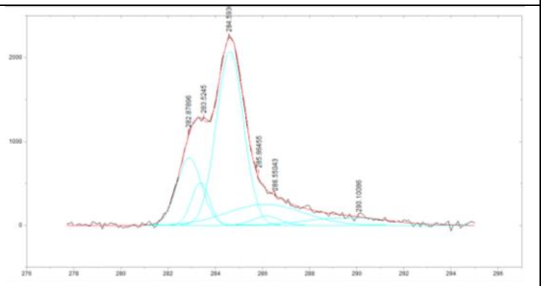

C-OH, COOH and π→π*	Not converged	A peak's intensity lying outside the spectrum at 290.16 eV	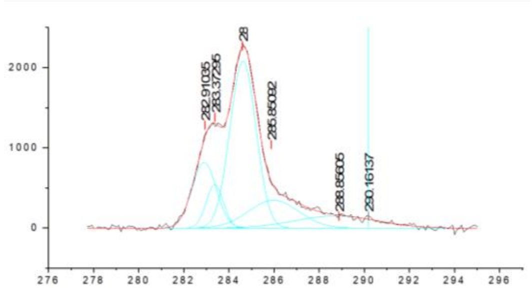
C-OH, C=O and π→π*	Not converged	A broad peak at 286.21 eV.<br><br>A peak's intensity shooting outside the spectra at 290.16 eV	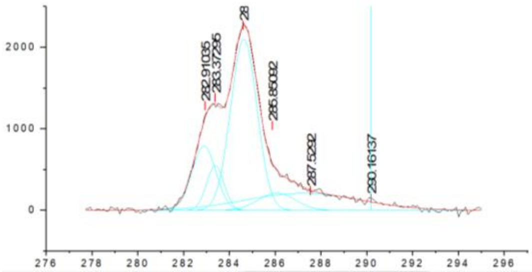
C-O-C, C=O and π→π*	Not converged	A peak with infinitely large FWHM at 285.94 eV	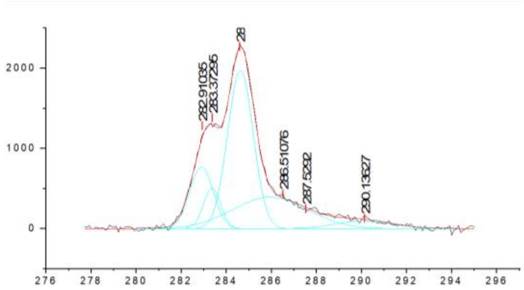
C=O, COOH and π→π*	Not converged	A broad peak at 286.50 eV<br><br>A peak outside the B.E. range (328.2 eV) with very large FWHM (77.86 eV)	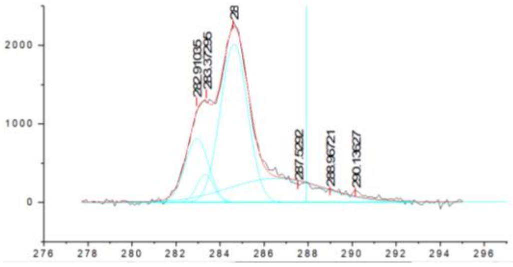
C-O-C, COOH and π→π*	99.83	A peak with very low FWHM (0.35 eV)	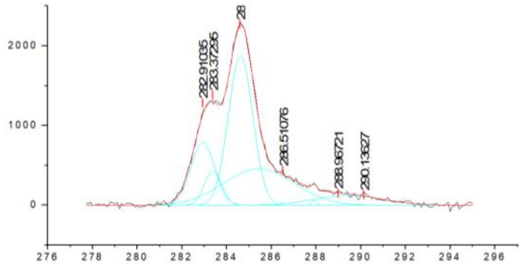

C-OH, C-O-C, COOH, and π→π*	99.82	Two peaks at same B.E (286.17 eV)<br>A broad peak at 289.6 eV (4.59)<br>Very low FWHM for π→π* transition (0.24 eV)	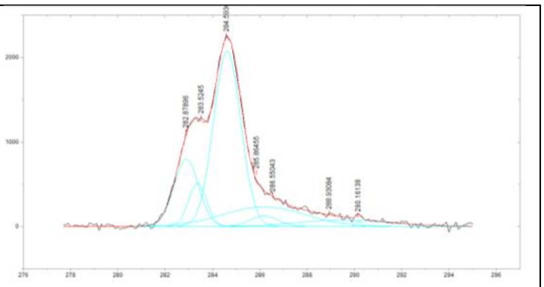
C-OH, C-O-C, C=O and π→π*	Not converged	A negative peak	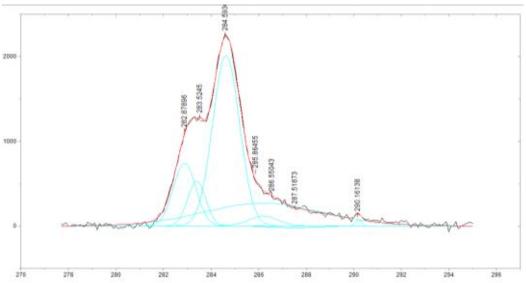
C-OH, C=O, COOH and π→π*	Not converged	A peak's intensity shooting outside the spectrum at 288.98 eV<br>A broad peak at 286.43 eV	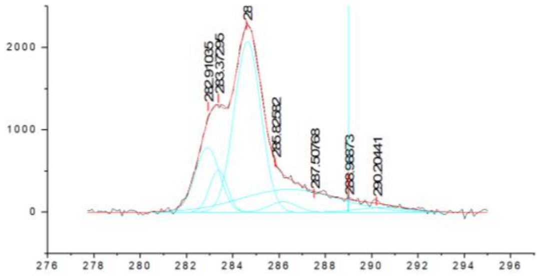
C-O-C, C=O, COOH and π→π*	Not converged	A negative peak at 288.72 eV	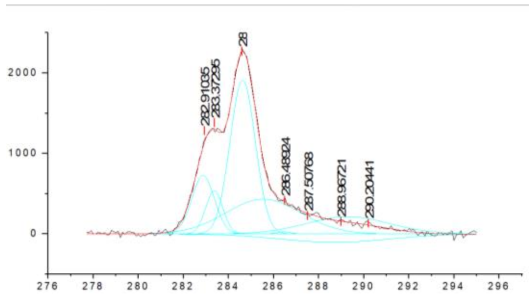
C-OH, C-O-C, C=O, COOH and π→π*	Not converged	A peak at infinite B.E.<br>Very low FWHM for C=O (0.26 eV)<br>Broad peak at 288.73 eV	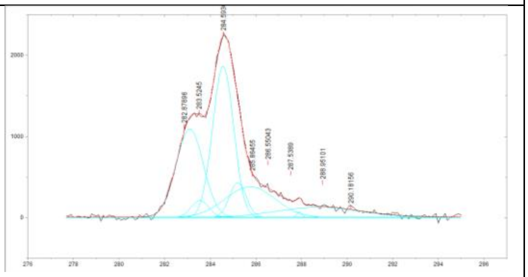

Table S8. Various trends of the cumulative fits obtained while fitting C-1s spectrum of *B/C*≈0.36.

**Functional groups**	**R^2**	**Remarks**	**Plot**
C-OH and π→π*	Not converged	A peak out of range<br>A broad peak at 286.08 eV	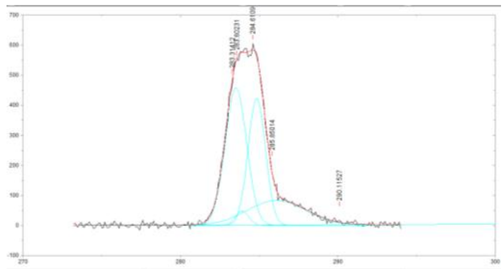
C-O-C and π→π*	99.78	A broad peak at 286.81 eV.	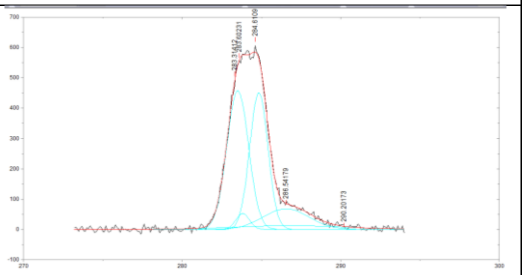
C=O and π→π*	99.78	A negative peak at 284.38 eV<br>infinitely large FWHM at 286.43 eV	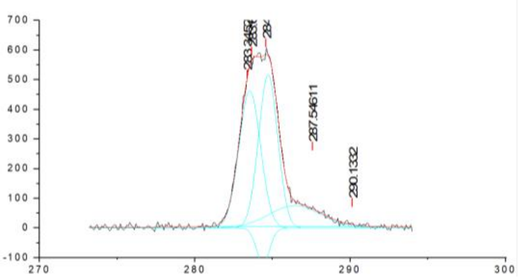
COOH and π→π*	Not converged	Infinitely large FWHM at 286.07 eV<br>Broad peak at 286.05 eV	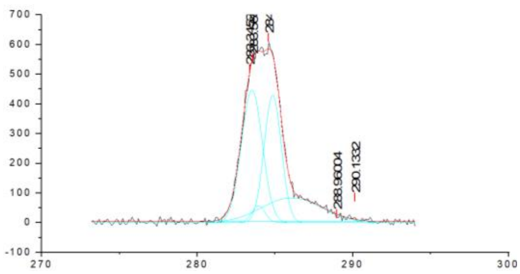

C-OH, C-O-C and π→π*	Not converged	A broad peak at 285.89 eV<br><br>A peak with infinite FWHM 286.48 eV<br><br>A negative peak at 286.48 eV	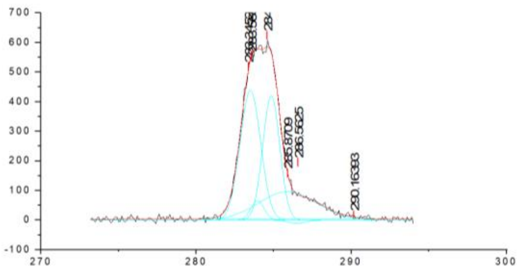
C-OH, C=O and π→π*	99.78	Two peaks at 285.93 eV:<br><br>One has very large FWHM (34.27 eV)<br><br>Other is a broad peak	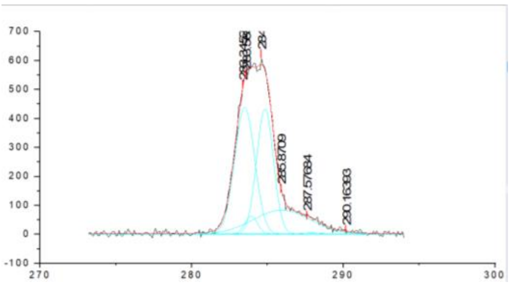
C-OH, COOH and π→π*	Not converged	A negative peak at 289.14 eV<br><br>Two peaks at 285.93 eV:<br><br>One has infinite FWHM<br><br>Other is broad peak	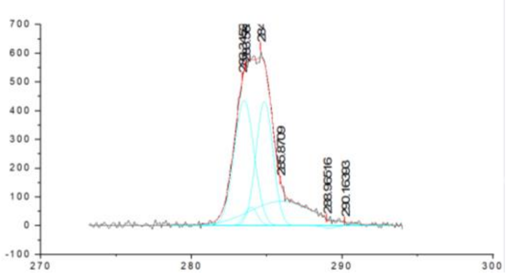
C-O-C, C=O, and π→π*	Not converged	Negative peak 284.96 eV with very low FWHM (0.006 eV)<br><br>Infinitely large FWHM for peak at 285.93 eV	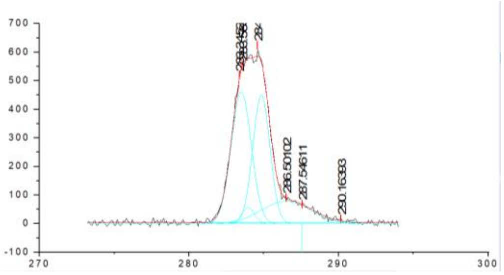

C-O-C, COOH and π→π*	Not converged	A negative peak 288.96 eV<br>A peak with infinite FWHM at 284.83 eV<br>An broad peak at 286.50 eV (4.96 eV)	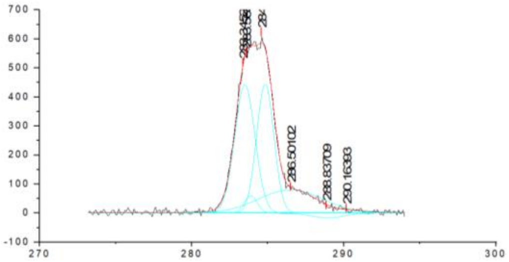
C=O, COOH and π→π*	Not converged	A negative peak at 288.54 eV.<br>Infinitely large FWHM at 284.94 eV<br>A broad peak at 287.51 eV (5.52 eV)	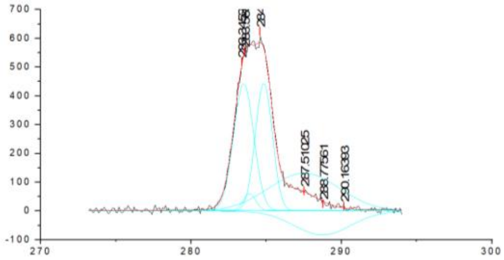
C-OH, C-O-C, C=O and π→π*	99.79	Two peaks at same binding energy 286.51 eV<br>One is a broad peak	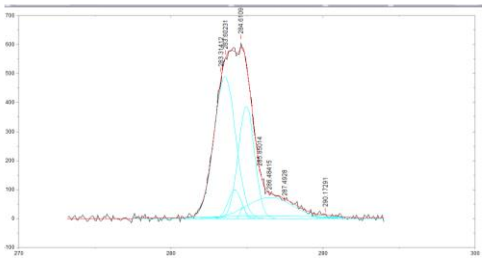
C-OH, C=O, COOH, and π→π*	Not converged	Negative peak at 284.42 eV<br>A peak with infinite intensity at 290.02 eV	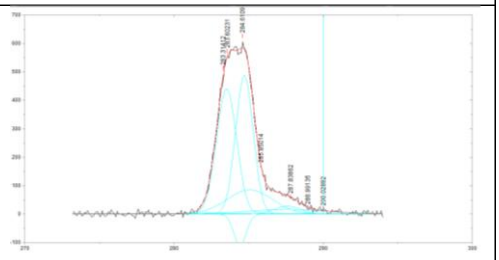
C-OH, C-O-C, COOH and π→π*	Not converged	A peak at 284.82 eV with infinitely large FWHM<br>A negative peak at 286.62 eV	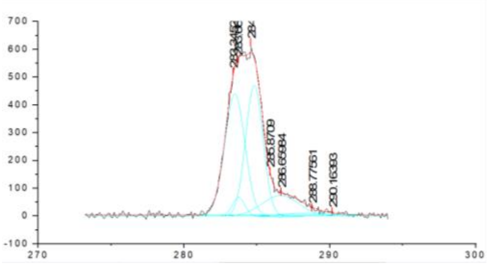

C-O-C, C=O, COOH and π→π*	Not converged	A Peak of infinite intensity at 290.2 eV<br><br>A peak of infinite FWHM at 290.2 eV<br><br>A negative peak at 284.35 eV	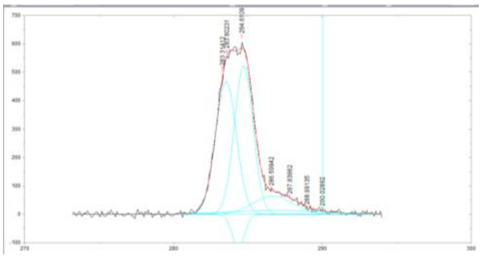
C-OH, C-O-C, C=O, COOH and π→π*	Not converged	Peak of infinite intensity at 290.2 eV<br><br>A peak of infinite FWHM at 290.2 eV<br><br>A negative peak at 284.35 eV	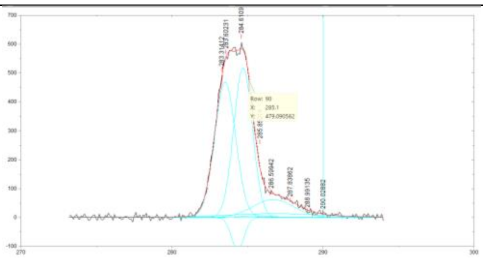

Table S9. Various trends of the cumulative fits obtained while fitting C-1s spectrum of *B/C*≈0.90.

**Functional groups**	**R^2**	**Remarks**	**Plot**
C-OH and π→π*	Not converged	Peak's intensity lying outside the spectrum at 290.11 eV<br><br>Broad peak at 285.03 eV	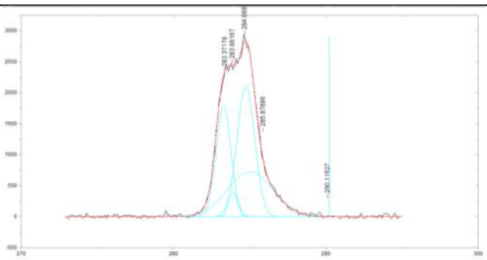
C-O-C and π→π*	99.84	Broad peak at 286.46 eV	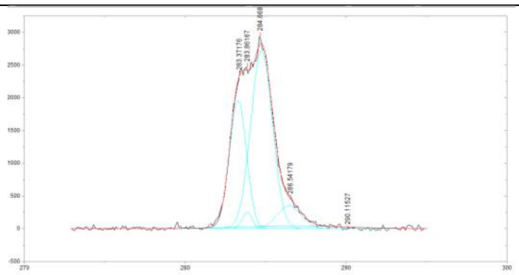

C=O and π→π*	Not converged	Very small FWHM at 290.12 eV (0.001 eV) and lying outside the spectrum<br><br>Negative peak at 284.38 eV<br><br>A broad peak at 285.14 eV	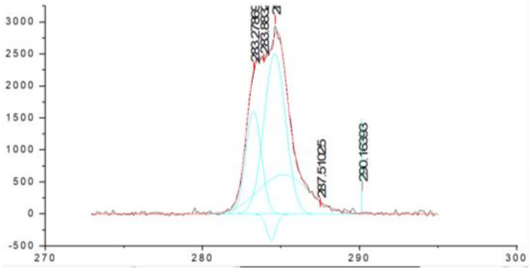
COOH and π→π*	Not converged	Negative peaks at 284.38 eV and 290.16 eV	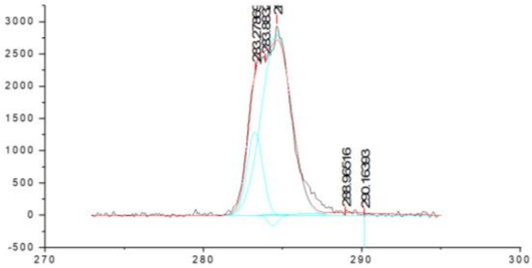
C-OH, C-O-C and π→π*	Not converged	3 negative peaks<br><br>A broad peak lying outside the spectrum	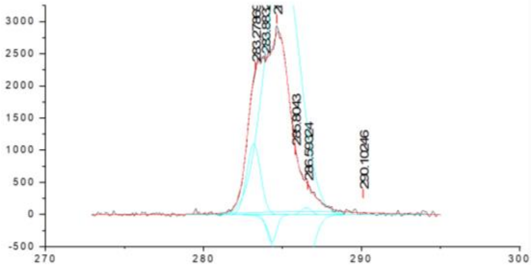
C-OH, C=O and π→π*	Not converged	3 negative peaks<br><br>A broad peak lying outside the spectrum	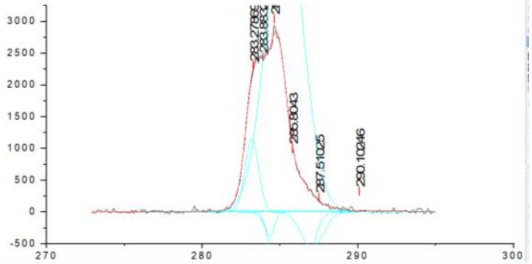
C-OH, COOH and π→π*	Not converged	3 negative peaks<br><br>A broad peak lying outside the spectrum	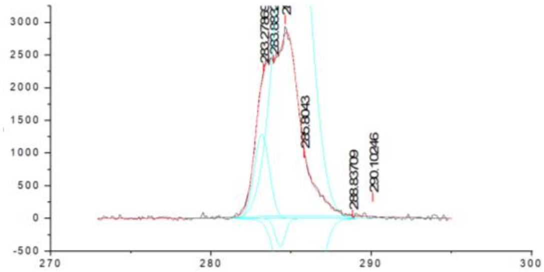

C-O-C, C=O and π→π*	Not converged	Very high FWHM (14.52 eV) at 286.96 eV<br><br>A negative peak at 284.33 eV	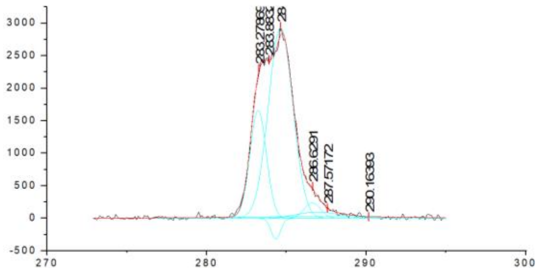
C-O-C, COOH and π→π*	Not converged	Very high FWHM (14.52 eV) at 286.96 eV.<br><br>A negative peak at 284.33 eV	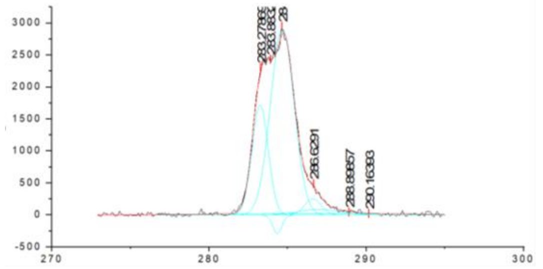
C=O, COOH and π→π*	Not converged	Negative peak of infinite intensity at 287.62 eV<br><br>A Peak of infinite intensity at 287.06 eV	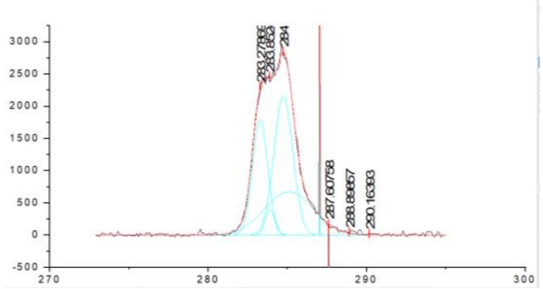
C-OH, C-O-C, C=O and π→π*	Not converged	Negative peaks at 284.30 eV and 285.78 eV<br><br>Broad peak at 287.62 eV	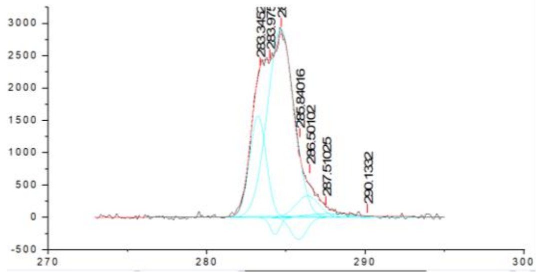
C-OH, C-O-C, COOH and π→π*	Not converged	Negative peaks at 284.25 and 285.76 eV<br><br>Large FWHM (34.16 eV) at 289.34 eV	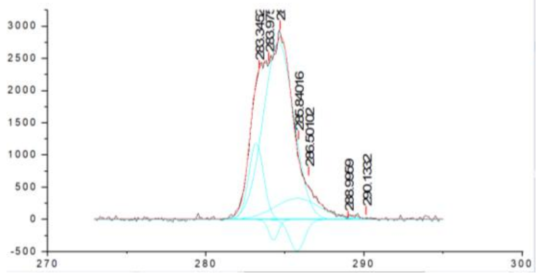
C-OH, C=O, COOH and π→π*	Not converged	Negative peaks at 284.25 and 285.76 eV<br><br>Infinitely large FWHM at 289.34 eV	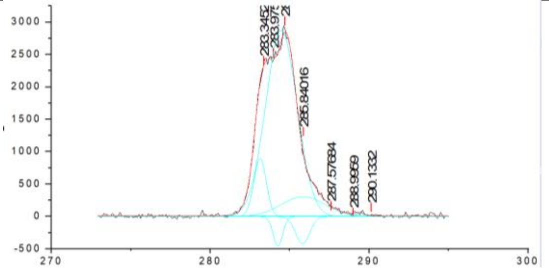

C-O-C, C=O, COOH and π→π*	Not converged	A peak with a B.E. lying outside the range of spectrum with infinitely large FWHM<br><br>A negative peak at 284.31 eV	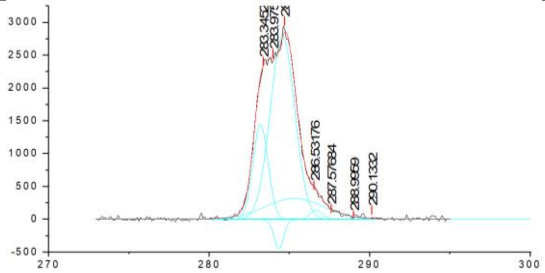
C-OH, C-O-C, C=O, COOH and π→π*	Not converged	A negative peak at 284.32 eV<br><br>A peak at infinite position	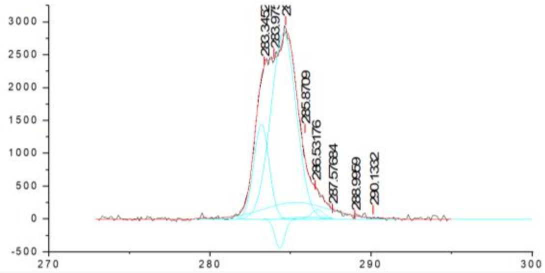

The C-1s core XPS spectra obtained from erGO are shown in Fig. S5. Peaks observed at binding energies ~284.44 - 284.55 eV, ~285.35 eV and 288.8 eV correspond to sp^2 hybridized C-C, C-OH (alkoxy), C-O-C (epoxy), and C=O (carbonyl) functional groups, respectively[9–11]. The oxygen functional groups are attached to C while erGO synthesis.

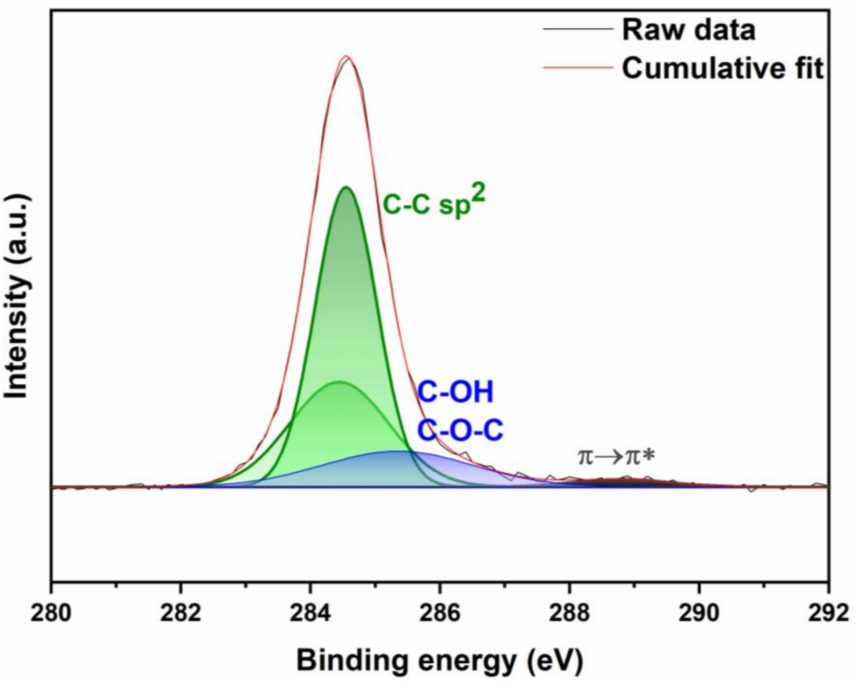

Figure S5. XPS C-1s core-level spectra obtained from erGO.

The B-1s core XPS spectra are shown in Fig. S6. The peaks corresponding to boron substituted carbon, B_4C, C_2-BO, C-BO_2 phases at ~189.1 eV, ~187.7 eV, ~191.4 eV, ~191.8 eV, respectively are absent[12].

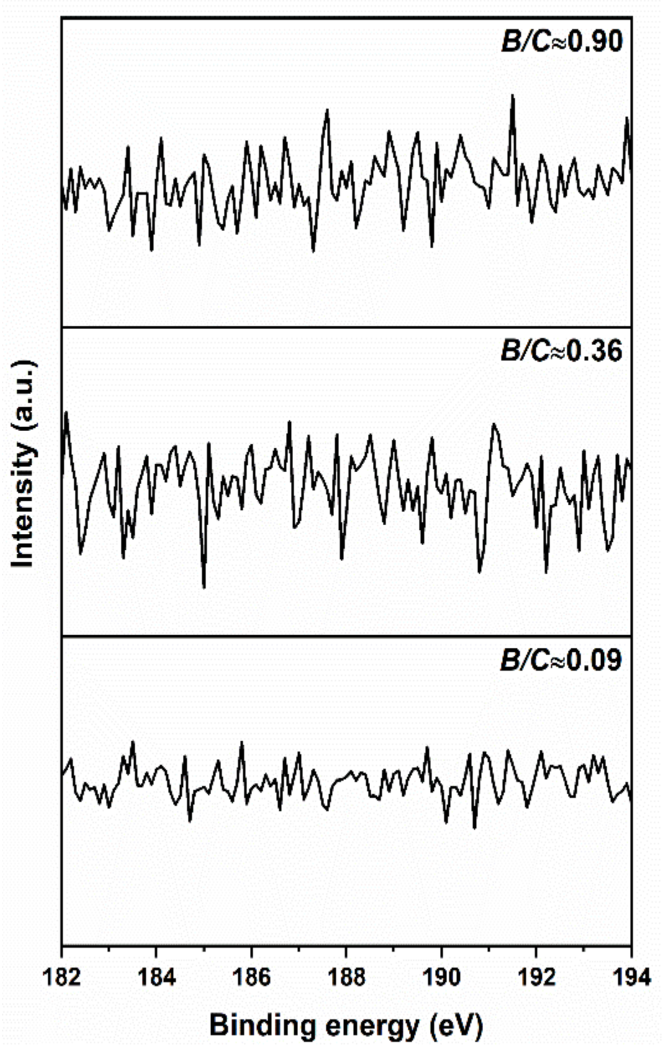

Figure S6. XPS B-1s core spectra for ball milled Mg-B-erGO nanocomposites at various *B/C* ratios.

S5. Composition of the elements used for ball milling

Table S10. Compositions of the elements used for synthesizing Mg-B-erGO nanocomposites.

B/C	**Mg (wt %)**	**erGO (wt %)**	**B (wt %)**	**B (at %)**
**0.00**	90	10	-	-
**0.09**	89.20	9.91	0.89	1.80
**0.36**	86.88	9.65	3.47	6.84
**0.90**	82.57	9.17	8.26	15.5

References

1. McCusker, L. B., Von Dreele, R. B., Cox, D. E., Louër, D. & Scardi, P. Rietveld refinement guidelines. *J. Appl. Crystallogr.* **32**, 36–50 (1999).

2. Rodrıguez-Carvajal, J. FULLPROF, a program for Rietveld refinement and pattern matching analyses. *Satellite Meeting on Powder Diffraction of the XVth Congress of the International Union of Crystallography* **127**, (1990).

3. Roisnel, T. & Rodríquez-Carvajal, J. WinPLOTR: a windows tool for powder diffraction pattern analysis. in *Materials Science Forum* **378**, 118–123 (Transtec Publications, 2001).

4. Young, R. A. & Wiles, D. B. Profile shape functions in Rietveld refinements. *J. Appl. Crystallogr.* **15**, 430–438 (1982).

5. Cullity, B. D. *Elements of X-ray Diffraction*. (Addison-Wesley Publishing, 1956).

6. Gull, S. F. & Daniell, G. J. Image reconstruction from incomplete and noisy data. *Nature* **272**, 686–690 (1978).

7. Smaalen, S. Van & Netzel, J. The maximum entropy method in accurate charge-density studies. *Phys. Scr.* **79**, 048304 (2009).

8. Major, G. H. *et al.* Practical guide for curve fitting in x-ray photoelectron spectroscopy. *J. Vac. Sci. Technol. A* **38**, 061203 (2020).

9. Li, S. *et al.* Plasma-induced highly efficient synthesis of boron doped reduced graphene oxide for supercapacitors. *Chem. Commun.* **52**, 10988–10991 (2016).

10. Rabchinskii, M. K. *et al.* From graphene oxide towards aminated graphene: facile synthesis, its structure and electronic properties. *Sci. Rep.* **10**, 1–12 (2020).

11. Kovtun, A. *et al.* Accurate chemical analysis of oxygenated graphene-based materials using X-ray photoelectron spectroscopy. *Carbon* **143**, 268–275 (2019).

12. Wang, G. *et al.* Interlayer coupling behaviors of boron doped multilayer graphene. *J. Phys. Chem. C* **121**, 26034–26043 (2017).
